# Oxidation of Aqueous Phosphorous Acid Electrolyte in Contact with Pt Studied by X-ray Photoemission Spectroscopy

**DOI:** 10.1021/acsami.3c12557

**Published:** 2023-10-27

**Authors:** Romualdus Enggar Wibowo, Raul Garcia-Diez, Tomas Bystron, Martin Prokop, Marianne van der Merwe, Mauricio D. Arce, Catalina E. Jiménez, Tzung-En Hsieh, Johannes Frisch, Alexander Steigert, Marco Favaro, David E. Starr, Regan G. Wilks, Karel Bouzek, Marcus Bär

**Affiliations:** † Dept. Interface Design, Helmholtz-Zentrum Berlin (HZB) für Materialien und Energie GmbH, Albert-Einstein-Str. 15, 12489 Berlin, Germany; ‡ Department of Inorganic Technology, University of Chemistry and Technology Prague, Technicka 5, Prague 6 166 28, Czech Republic; § Departamento Caracterización de Materiales, INN-CNEA-CONICET, Centro Atómico Bariloche, Av. Bustillo 9500, S. C. de Bariloche, Rio Negro 8400, Argentina; ∥ Energy Materials In-situ Laboratory Berlin (EMIL), HZB, Albert-Einstein-Str. 15, 12489 Berlin, Germany; ⊥ Institute for Nanospectroscopy, Helmholtz-Zentrum Berlin für Materialien und Energie GmbH (HZB), Albert-Einstein-Str. 15, 12489Berlin,Germany; # Institute for Solar Fuels, Helmholtz-Zentrum Berlin für Materialien und Energie GmbH (HZB), Hahn-Meitner-Platz 1, 14109Berlin, Germany; ∇ Department of Chemistry and Pharmacy, Friedrich-Alexander-Universität Erlangen-Nürnberg (FAU), Egerlandstr. 3, 91058 Erlangen, Germany; ○ Department of X-ray Spectroscopy at Interfaces of Thin Films, Helmholtz Institute Erlangen-Nürnberg for Renewable Energy (HI ERN), Albert-Einstein-Str. 15, 12489 Berlin, Germany

**Keywords:** Pt/H_3_PO_3_ interaction, aqueous H_3_PO_3_ oxidation, ion-exchange chromatography, *in situ* AP-HAXPES, Pt|aqueous H_3_PO_3_ interface, HT-PEMFCs

## Abstract

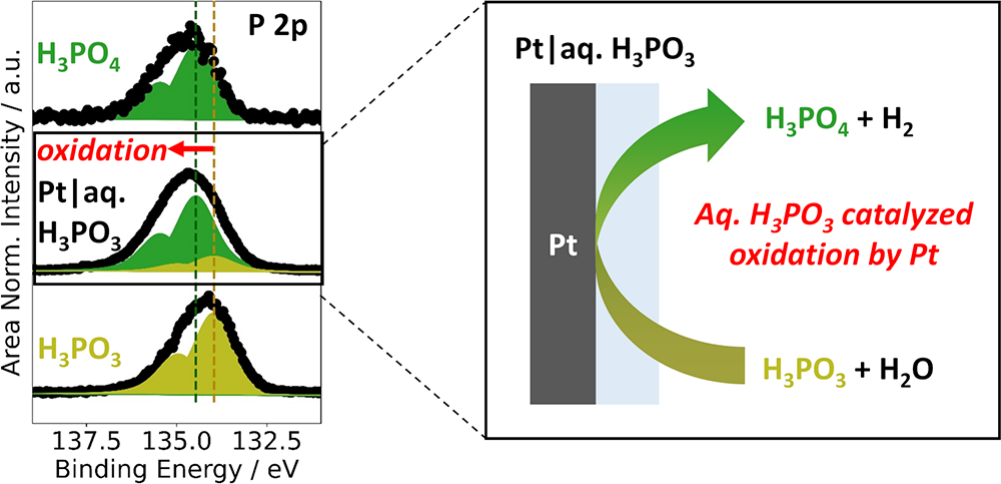

The oxidation of the aqueous H_3_PO_3_ in contact with Pt was investigated for a fundamental understanding of the Pt/aqueous H_3_PO_3_ interaction with the goal of providing a comprehensive basis for the further optimization of high-temperature polymer electrolyte membrane fuel cells (HT-PEMFCs). Ion-exchange chromatography (IEC) experiments suggested that in ambient conditions, Pt catalyzes H_3_PO_3_ oxidation to H_3_PO_4_ with H_2_O. X-ray photoelectron spectroscopy (XPS) on different substrates, including Au and Pt, previously treated in H_3_PO_3_ solutions was conducted to determine the catalytic abilities of selected metals toward H_3_PO_3_ oxidation. *In situ* ambient pressure hard X-ray photoelectron spectroscopy (AP-HAXPES) combined with the “dip-and-pull” method was performed to investigate the state of H_3_PO_3_ at the Pt|H_3_PO_3_ interface and in the bulk solution. It was shown that whereas H_3_PO_3_ remains stable in the bulk solution, the catalyzed oxidation of H_3_PO_3_ by H_2_O to H_3_PO_4_ accompanied by H_2_ generation occurs in contact with the Pt surface. This catalytic process likely involves H_3_PO_3_ adsorption at the Pt surface in a highly reactive pyramidal tautomeric configuration.

## 1 Introduction

Hydrogen-operated high-temperature polymer electrolyte membrane fuel cells (HT-PEMFCs) with H_3_PO_4_-doped membranes represent an attractive option as a stationary energy source. HT-PEMFCs have not only a high heat and power conversion efficiency^1,2^ but also possess multiple advantages over their low-temperature counterparts due to the elevated operating temperatures (120–180 °C). These advantages include higher tolerance toward CO poisoning,^3−5^ cogeneration of electricity and heat, and also potential operation when coupled to a reformer system,^6−8^ allowing for a feed of liquid or other gaseous fuels. While the use of a H_3_PO_4_-doped membrane provides the possibility of high-temperature use with the aforementioned advantages, it also brings several disadvantages, the majority of which are related to H_3_PO_4_ leaching out of the membrane. Additionally, numerous studies showed the poisoning of Pt by H_3_PO_4_ and its anion (e.g., H_2_PO_4_^–^),^9,10^ which negatively impacts the kinetics of the oxygen reduction reaction and decreases the performance of the fuel cell. Furthermore, several investigations indicated a reduction of H_3_PO_4_ leading to the production of other phosphorus-containing compounds (e.g., H_3_PO_3_) during the operation of the HT-PEMFC.^11−14^

The formation of phosphorus compounds with phosphorus in an oxidation state lower than +5 has been associated with the direct electrochemical reduction of H_3_PO_4_ as well as the chemical reduction of H_3_PO_4_ in the presence of H_2_, catalyzed by Pt.^14^ In previous works by Prokop et al., it was shown that the corresponding reduction products adsorb on the Pt surface, limiting the adsorption of H_2_ and O_2_ on the Pt electrode.^14,15^ In particular, H_3_PO_3_ was strongly adsorbed on the bare Pt surface and, in contact with PtO_*x*_, chemical oxidation of H_3_PO_3_ to H_3_PO_4_ occurred.^15^ The surface coverage of H_3_PO_3_ on Pt has also been studied.^15^ Its unusual temperature dependence was explained by considering tautomeric equilibria between the thermodynamically more stable inactive tetrahedral form and the more active but much less stable pyramidal form. In particular, the pyramidal H_3_PO_3_ form adsorbs strongly on the Pt surface and the degree of its adsorption increases with rising temperature,^15^ which is interesting from the point of view of practical HT-PEMFC applications, operating usually at 120–180 °C. Recently, it was shown that H_3_PO_3_ adsorbs strongly also on nanoparticulate Pt, which negatively affects the electrochemically active surface area and kinetics of the O_2_ reduction reaction, particularly in the high current density region.^16^ Moreover, a recent study showed, through a combination of X-ray absorption spectroscopy at the Pt *L*_3_-edge and density functional theory, that H_3_PO_3_ is more strongly adsorbed on Pt compared to H_3_PO_4_.^17^ In HT-PEMFCs, although the concentration of H_3_PO_3_ is low, as it exists only as impurities in concentrated H_3_PO_4_ electrolyte, these studies indicate that H_3_PO_3_ could poison the Pt catalyst and therefore negatively impact the performance of the HT-PEMFCs. Furthermore, it is plausible that the detrimental effect of H_3_PO_4_ on the kinetics of Pt electrode observed in the precedent studies^10,18,19^ might be partially attributed to the H_3_PO_3_ formed by the reduction of H_3_PO_4_. Consequently, thorough investigations of H_3_PO_3_ interaction with Pt and its oxidation behavior on Pt are necessary for further optimization of HT-PEMFCs. However, currently there is a lack of investigation on the oxidation behavior of H_3_PO_3_ on precious metal electrodes, especially on Pt.

In this study, we aim to investigate the interaction of H_3_PO_3_ with Pt under open circuit potential (OCP) conditions (i.e., without external polarization). Ion exchange chromatography (IEC), X-ray photoelectron spectroscopy (XPS) measurements of aqueous H_3_PO_3_ (with/without contact to Pt), and *in situ* ambient pressure hard X-ray photoelectron spectroscopy (AP-HAXPES) of the Pt|H_3_PO_3_ electrolyte interface were performed, to shed light on the oxidation mechanism of H_3_PO_3_ on Pt. Additionally, electrochemical characterizations by cyclic voltammogram (CV) on an aqueous H_3_PO_3_-based solution with Pt electrodes were performed to provide complementary information regarding the interaction of Pt and H_3_PO_3_. Detailed insights into the fundamental mechanism of the H_3_PO_3_ interaction with Pt are revealed, a prerequisite for more complex (e.g., *operando*) studies.

## 2 Experimental Section

### a IEC Analysis of Aqueous H_3_PO_3_ Solutions

An aqueous solution of H_3_PO_3_ (98 wt %, extra pure, Acros Organics) with a concentration of 10 mmol dm^–3^ was prepared using deionized water from DIWA purifier (conductivity <0.5 μS m^–1^, WATEK). Fifteen cm^3^ of the solution was deaerated with N_2_ (99.995 vol %, SIAD) for 30 min and then kept in a sealed glass container.

In the first experiment, 50 mg of Pt/C catalyst HiSPEC4000 (40 wt % Pt, Johnson-Matthey) was added to 15 cm^3^ of deaerated solution at 25 °C. Stirring at 1000 rpm using PTFE-sealed magnetic stirrer was maintained throughout the experiment.

The second experiment used an electrochemical cell filled with 80 cm^3^ of the aforementioned deaerated H_3_PO_3_ solution in a two-electrode setup, with a working gas diffusion electrode (area 2 × 3 cm^2^, containing 7.5 mg of Pt/C catalyst), and Hg|Hg_2_SO_4_|K_2_SO_4(sat.)_ reference electrode separated from the electrolyte by a double liquid junction. During the experiment, the electrolyte was vigorously stirred as in the first experiment and constantly bubbled with N_2_. The open circuit potential (OCP) was recorded using a PARSTAT MC potentiostat (AMETEK) and recalculated versus a standard hydrogen electrode (SHE). The gas diffusion electrode was made in-house using ultrasonically assisted deposition of ink, containing Pt/C catalyst and PTFE binder Algoflon F5 (Solvay) in water and isopropanol 1:1, on carbon paper Sigracet 38BC (SGL). After deposition, the electrode was heat-treated under an inert atmosphere at 350 °C for 30 min. The Pt loading on the electrode was 0.5 mg cm^–2^, and the binder content in the catalyst layer was 10 wt %.

At selected time intervals during both experiments, 1 cm^3^ of the solution was extracted for IEC using a syringe with a membrane filter (CHROMAFIL O-20/15MS, MACHEREY-NAGEL). Subsequently, the sample and experimental solution were deaerated for 30 s. All of the experiments were performed at room temperature.

Deaerated samples of 1 cm^3^ volume were analyzed on Dionex Integrion HPIC with a hydroxide-selective anion-exchange precolumn Dionex IonPac AS19–4 μm (2 × 50 mm) IC and column Dionex IonPacTM AS19–4 μm (2 × 250 mm) with anion dynamic self-regulating suppressor ADRS 600, auto sampler AS-AP and conductivity detector CR-ATC 600 for analysis of inorganic anions (Thermo Scientific). The injected sample volume was 0.025 cm^3^. The mobile phase was either a 20 or 15 mmol dm^–3^ KOH solution with a volumetric flow rate of 0.25 cm^3^ min^–1^. Suppressor currents were 13 and 10 mA for 20 and 15 mmol dm^–3^ KOH solutions, respectively. The eluent was generated by automated mixing of KOH solution from a cartridge (EGC 500 KOH) with demineralized water. To determine the concentration of H_3_PO_3_ and H_3_PO_4_ in the samples, a calibration curve for the IEC peak area was obtained with standard solutions, prepared using deionized water, H_3_PO_3_, and H_3_PO_4_ (85 wt %, extra pure, Acros Organics).

### b XPS of “Acid-Treated Metal Electrodes” and Electrochemical Characterization of the Planar Electrode

Au foil (99.95%, Alfa Aesar), Pt foil (99.95%, Goodfellow), and a Pt black electrode were used in the study. Planar Pt foil and Au foil (which will be termed “planar Pt” and “planar Au” in the following discussion) were used in the study to minimize the possible influence of surface roughness on the oxidation of H_3_PO_3_. Therefore, any difference in the observed results between the planar electrodes will depend on the distinct interaction between H_3_PO_3_ and the metal. To affirm that both planar metal electrodes used in the study possess low and comparable surface roughness, the surface roughness was determined by using atomic force microscopy, AFM (Park Systems, XE-70). From the AFM it was determined that the planar Au and planar Pt have an average surface roughness of (3.5 ± 1.2) nm and (3.5 ± 0.6) nm, respectively. Additionally, the roughness factor was also estimated from the electrochemically active surface area (ECSA) of the electrodes, determined from hydrogen underpotential deposition (H_UPD_) and copper underpotential deposition (Cu_UPD_), for the Pt and Au electrodes, respectively, in which both electrodes also display comparable roughness factors. AFM scans and the ECSA determination for both planar metal electrodes are given in Supporting Information (SI), Figure S1. Furthermore, a rougher Pt black electrode with a higher surface area than planar Pt (which will be referred to as “Pt black” in the following discussion), was used to gain insight into the influence of the Pt surface roughness on the oxidation of H_3_PO_3_ (by comparing observation from planar Pt and Pt black) and as reference for the *in situ* AP-HAXPES experiments described in Section 2.3.

To prepare the Pt black, first: a planar Pt film was prepared by sputtering a 15 nm-thick Pt layer on a Si wafer. Sputtering was performed in the DC magnetron mode (PREVAC) with a process pressure of 4 × 10^–3^ mbar (base pressure was 1 × 10^–8^ mbar), using Ar as working gas, and a sputter rate of ∼5 nm/min. The sputter power was set at 50 W on a 2 in. Pt target. Following the sputtering process, the sputtered Pt electrode was electrochemically cleaned in N_2_-saturated 0.5 mol dm^–3^ H_2_SO_4_ (prepared by diluting 95 wt % H_2_SO_4_ (Merck), with Milli-Q water (Q-POD, conductivity ∼0.055 μS cm^–1^)) by potential cycling within the water stability window: + 0.05 V to +1.0 V vs RHE with a scan rate of 50 mV s^–1^, until a steady-state voltammogram was obtained. Subsequently, Pt black was electrodeposited on top of the planar nonporous Pt surface, creating a rough-high-surface area Pt. Electrodepositions were conducted by using 2 mol dm^–3^ HCl (prepared by diluting 37 wt % HCl, Carl Roth, with Milli-Q water) + 2 wt % H_2_PtCl_6_ (99.9%, Alfa Aesar) solution in a two-electrode setup with a Pt mesh (99.9%, Alfa Aesar) as a counter electrode. A current density of −10 mA cm^–2^ was applied to the working electrode for 10 min to perform the Pt black electrodeposition process. As a confirmation of the desired surface morphology, scanning electron microscopy (ZEISS, MERLIN) images of the planar Pt and the Pt black electrodes are included in Figure S2 in the SI.

For the electrolyte, H_3_PO_3_ and H_3_PO_4_ acid solutions of 5 mol dm^–3^ concentration were prepared by diluting either H_3_PO_3_ (99 wt %, Merck) or crystalline H_3_PO_4_ (99.99 wt %, Merck) in Milli-Q water. The concentration of 5 mol dm^–3^ was used to keep the conditions similar to the *in situ* AP-HAXPES measurement. The solutions were degassed by storing the solution inside a dedicated chamber and evacuating it to the pressure of ∼15 mbar for around 30 min. The degassing was performed in order to minimize the contribution of O_2_ to the investigation.

After degassing, the solution was kept under an inert atmosphere (in an Ar-purged glovebag). Subsequently, the Pt and Au electrodes were immersed in the solution for about 60 s. The electrodes were dried by softly flushing them with Ar and subsequent evacuation (for approximately 2.5 h) in the transfer chamber allowing a direct transfer from the glovebox into the ultrahigh vacuum (UHV) system of the XPS analysis system. In the following discussion, these electrodes will be called “H_3_PO_3_-treated electrodes” and “H_3_PO_4_-treated electrodes”, for electrodes that have previously immersed in the 5 mol dm^–3^ H_3_PO_3_ electrolyte and 5 mol dm^–3^ H_3_PO_4_ electrolyte, respectively. The XPS measurements were conducted with Mg Kα excitation (1253.56 eV, Specs XR 50) and a hemispherical electron energy analyzer (ScientaOmicron Argus CU electron analyzer), using a pass energy of 20 eV. The base pressure of the XPS system was <5 × 10^–8^ mbar. The binding energy was calibrated either to the Au 4f_7/2_ peak of grounded Au foil at 84.00 eV, or to the Pt 4f_7/2_ core level of grounded Pt foil at 71.00 eV.^20^

For an insight into the electrochemical behavior of H_3_PO_3_ in contact with these electrodes, two electrochemical characterization methods were performed: (i) OCP recording using the aforementioned working electrodes (Pt black, planar Pt, and planar Au) in a N_2_ saturated 5 mol dm^–3^ H_3_PO_3_ under constant stirring (380 rpm, using IKA C-MAG HS7 magnetic stirrer) and (ii) cyclic voltammograms with the planar metal working electrodes (i.e., planar Pt and planar Au) also in a N_2_ saturated 5 mol dm^–3^ H_3_PO_3_. These experiments were carried out with a Pt mesh counter electrode (99.9%, Alfa Aesar) and reversible hydrogen reference electrode (Mini HydroFlex, Gaskatel), by using a BioLogic SP-300 double channel potentiostat. Prior to the electrochemical characterization, the H_3_PO_3_ electrolyte was prepared and degassed with the aforementioned method. For comparison, cyclic voltammetry measurements were conducted with 5 mol dm^–3^ H_3_PO_4_. The recorded current was normalized by the geometrical area of the electrode in contact with the electrolyte (given in Table S1 of the SI). Further details on the OCP monitoring experiments can be found in section S6 of the SI.

### c *In Situ* AP-HAXPES of the Pt|H_3_PO_3_ Interface and the Pt|H_3_PO_4_ Interface

A set of H_3_PO_3_ and H_3_PO_4_ solutions, with concentrations of 1 and 5 mol dm^–3^ was prepared according to the procedure described in Section 2.2. These relatively high concentrations were used to maximize the signal-to-noise ratio while minimizing the acquisition time for the *in situ* AP-HAXPES measurements (i.e., the time to record high-quality P 2p core level spectra) at the electrode|thin electrolyte interface (i.e., to prevent/minimize a possibility of synchrotron-radiation-induced beam damage in a prolonged measurement duration). Two different concentrations were used to observe the influence of H_2_O on the oxidation of H_3_PO_3_. Prior to the experiment, the electrolyte was degassed in a dedicated chamber with a pressure of ∼10 to 15 mbar for around 1.5 h. Pt black prepared according to the procedure given in Section 2.2 was used as a working electrode, as it provides a higher surface area compared to a planar Pt electrode prepared by sputtering. The increased wettability of the Pt black electrode compared to nonporous sputtered Pt enabled an easier formation of the necessary thin film electrolyte during the “dip-and-pull” procedure required to allow probing of the Pt|H_3_PO_3_ interface by *in situ* AP-HAXPES.

*In situ* AP-HAXPES experiments were conducted in the SpAnTeX end-station^21^ located at the bending magnet beamline KMC-1 of BESSY II^22^ with the aforementioned solutions, Pt black as the working electrode, Pt foil as the counter electrode (99.95%, Goodfellow), and a miniaturized Ag|AgCl|KCl_(sat.)_ reference electrode (ET072, eDAQ). The solid/liquid interface was created using the “Dip-and-pull” method.^23−25^ The incoming X-rays were monochromatized using the Si (111) crystal pair of a double crystal monochromator and kept at an energy of 3 keV. The measurements were conducted with a beam spot of approximately 400 μm × 700 μm.^21^ Spectra were recorded using a hemispherical electron energy analyzer (PHOIBOS 150 HV NAP, SPECS), with a pass energy of 20 eV. The binding energy (BE) scale was calibrated to the Au 4f_7/2_ core level of Au foil, which was mounted in the vicinity of the Pt black electrode, at a BE of 84.00 eV.^20^ Around 1.5 h of measurement time was required for the acquisition of spectra with an adequate signal-to-noise ratio of the core level of interest (O 1s, Pt 4f, and P 2p). The pressure in the analysis chamber was between 18 and 22 mbar for all measurements.

For the electrochemical characterizations conducted during the *in situ* “dip-and-pull” experiments (e.g., cyclic voltammetry), the recorded currents were normalized by the geometrical area of the electrode, estimated from the length of the electrode that has been dipped in the electrolyte. In these experiments, a common ground was shared between the electron energy analyzer and the potentiostat used for electrochemical measurements (BioLogic SP-300 double channel). Further details on the electronic connection between the potentiostat and the analyzer can be found in ref (21).

### d AP-HAXPES on Crystalline H_3_PO_3_ and Crystalline H_3_PO_4_ Solid Reference

As references of H_3_PO_3_ and H_3_PO_4_ in the pristine state, AP-HAXPES was conducted on solid crystalline H_3_PO_3_ (99 wt %, Merck) and solid crystalline H_3_PO_4_ (99.99 wt %, Merck), which will be referred to as H_3_PO_3_ reference and H_3_PO_4_ reference in the following discussion. These references were mounted on carbon tape in an inert atmosphere for subsequent AP-HAXPES measurements.

For the AP-HAXPES measurement, the pressure was kept at 5 mbar. Only reference compounds were placed in the analysis chamber, no additional gases were flown to the analysis chamber. Experiments were made in this pressure to keep the measurement pressure higher than the vapor pressure of the compounds (∼3 mbar), thus avoiding possible changes to the solid crystalline acids’ properties, as well as to mitigate a possible charging effect in the HAXPES measurement.^26^

Similar to the “dip-and-pull” AP-HAXPES measurements, these experiments were made in the SpAnTeX end-station^21^ located at the KMC-1 beamline^22^ of BESSY II. A tender monochromatized X-ray of 2.12 keV excitation was used to probe these solid crystalline acids. For the energy calibration, the Au 4f_7/2_ core level of a clean Au foil was measured, then the binding energy was set to 84.00 eV.^20^

### e XPS Curve Fit Analysis

For the XPS analysis on the P 2p core level, two Voigt profiles, corresponding to P 2p_3/2_ and P 2p_1/2_, as well as a Shirley background were used for the fit model of each solid H_3_PO_3_ reference and H_3_PO_4_ reference. For a physically meaningful fit, the fwhm (full-width half-maximum) of the P 2p_3/2_ components were kept similar to P 2p_1/2_ components. In addition, the peak area of P 2p_1/2_ was constrained to 50% of P 2p_3/2_ component.^27^ Subsequently, fitting was conducted on the spectra of the acid-treated electrodes as well as the *in situ* AP-HAXPES. With the assumption that the XPS results in the acid-treated electrode and *in situ* AP-HAXPES emerge from H_3_PO_3_ and H_3_PO_4_-like compounds, a similar fitting parameter from solid H_3_PO_3_/H_3_PO_4_ references was used, since they should contain properties similar to those of the solid crystalline acid references (shown in Table S2 in the SI). Specifically, the following fitting procedure was used: binding energy of peak maxima and fwhm of each H_3_PO_3_ and H_3_PO_4_ component for both acid-treated & *in situ* AP-HAXPES results were kept to the same value as the solid references given in Table S2, while peak amplitude (i.e., area) was allowed to vary. Furthermore, since several papers reported varying peak separation of P 2p_3/2_ and P 2p_1/2_, between 0.7 and 1 eV,^28−31^ doublet separation was optimized within this region, and then was kept constant for the final fitting on all compounds. The fitting process was performed by using the Python code built on the LMFIT package.^32^ Final fitting parameters for the solid references, the acid-treated electrodes, and the *in situ* AP-HAXPES, are given in Table S2, S3, and S7 in the SI, respectively.

## 3 Results and Discussion

**Figure 1:**
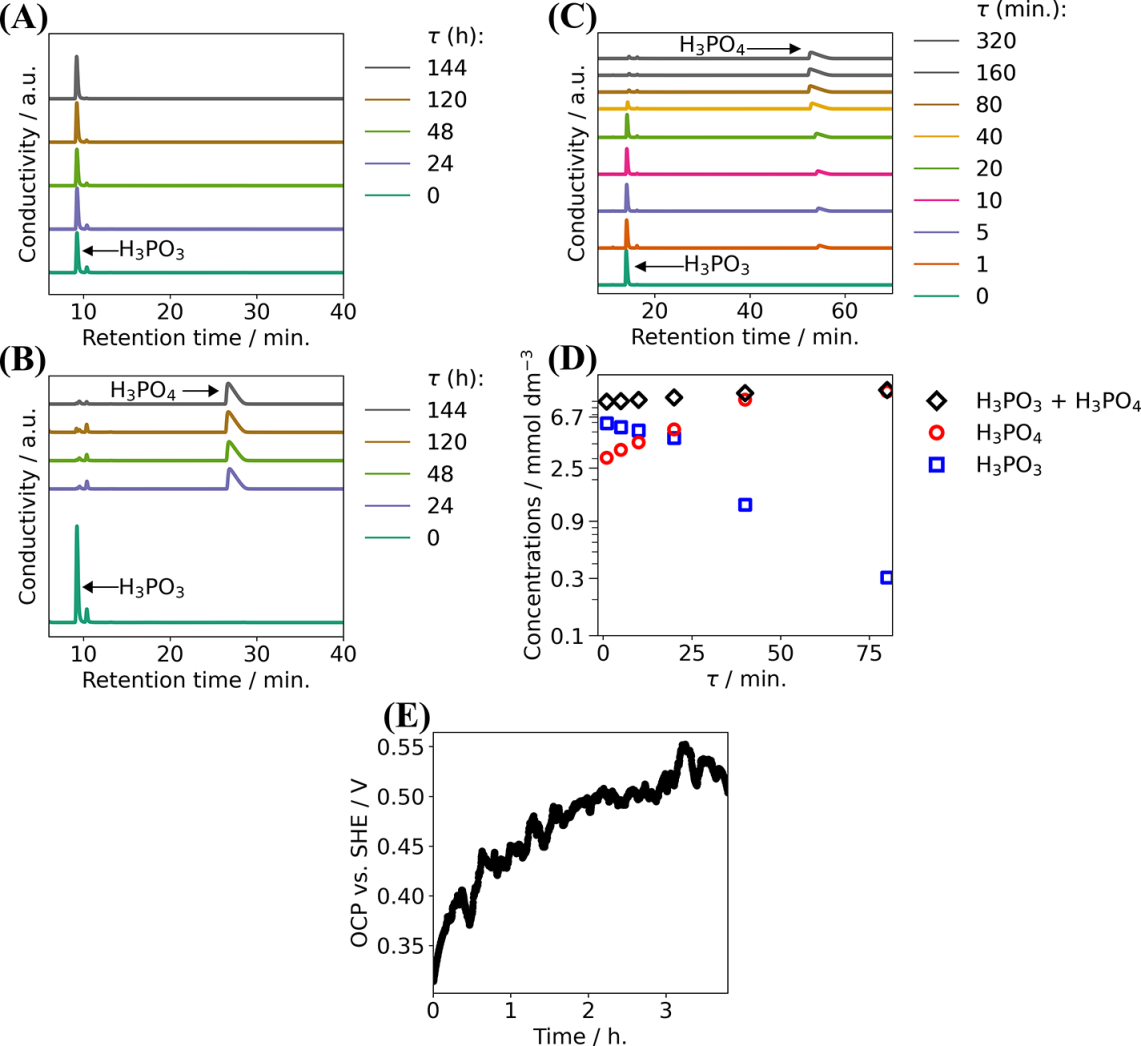
Results of IEC analysis of a 10 mmol dm^–3^ H_3_PO_3_ solution (total volume of 15 cm^3^). IEC chromatograms measured (A) in the absence of Pt/C and (B, C) with 50 mg of Pt/C catalyst (40 wt % Pt) with the corresponding concentration profile of H_3_PO_3_ and H_3_PO_4_ monitored on a larger (B) and shorter (C) time scale. (D) Concentration of H_3_PO_3_ and H_3_PO_4_ extracted from panel (C) as a function of time. Eluent solution in panels (A) and (B) was 20 mmol dm^–3^ KOH, and eluent in panel (C) was 15 mmol dm^–3^ KOH. In water, H_3_PO_3_ is stable for 144 h, but upon the addition of Pt/C, there is a conversion of H_3_PO_3_ to H_3_PO_4_ in a very short time scale. (E) Recording of OCP during the experiment with a gas diffusion electrode (total content of Pt/C: 7.5 mg, area 2 × 3 cm^2^, electrolyte volume 80 cm^3^).

Since the stability of an aqueous H_3_PO_3_ solution (which is thermodynamically unstable) is a key parameter for further analyses, IEC analysis experiments were made to monitor the H_3_PO_3_ concentration in the aqueous electrolyte in the absence of O_2_ (see Figure 1). To establish the retention time of the species of interest, several chemical standards were used for calibration. Based on the calibration within the concentration range 1–100 ppm, the retention time of HPO_3_^2–^ was around 9 min. At a slightly longer retention time (around 12 min.) (Figure 1A,B), traces of NO_2_^–^ and NO_3_^–^ were observed as well, likely due to contamination of the column. The retention time of HPO_4_^2–^/PO_4_^3–^ was around 27 min and the peak has a specific, deformed shape (pH of the mobile phase is very close to p*K*_a,3_ value of H_3_PO_4_). This wide gap between retention times of HPO_3_^2–^ and HPO_4_^2–^/PO_4_^3–^ enabled a very precise determination of both compounds’ concentrations. As shown from the results presented in Figure 1A, the H_3_PO_3_ concentration in the bare aqueous solution remained unchanged for the entire experiment (144 h). However, when the same experiment was performed with Pt/C powder (50 mg) added into the solution (15 cm^3^), practically no H_3_PO_3_ was found in the solution after 24 h and it was clearly converted to H_3_PO_4_, see Figure 1B. Therefore, the experiment with Pt/C powder added into the H_3_PO_3_ solution was repeated and samples were taken in shorter periods allowing better time resolution (Figure 1C). To further improve the peak separation, the concentration of KOH in the mobile phase was decreased to 15 mmol dm^–3^ in this experimental series, leading to a shift of retention times to higher values. The last experiment was performed in an electrochemical cell by using a gas diffusion electrode (GDE) and reference electrode for the OCP recording (Figure 1E).

These experiments revealed that H_3_PO_3_ in water is stable for at least 144 h (Figure 1A). However, a significant amount of H_3_PO_3_ was converted to H_3_PO_4_ within a few minutes after Pt/C catalyst addition, shown in Figure 1C,D. The total concentration of H_3_PO_3_ + H_3_PO_4_ remains constant, as is evident from the concentration profiles in Figure 1D, determined on the basis of chromatograms presented in Figure 1C. This states that Pt catalyzes the oxidation of H_3_PO_3_ to H_3_PO_4_ by water (see eq 1) involving half reactions eq 2 and eq 3.
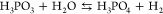
1
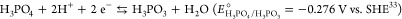
2
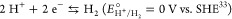
3

Furthermore, the electrochemical cell experiment (Figure 1E) indicates that oxidation of H_3_PO_3_ occurred through the process shown in eq 1. This experiment revealed that OCP slowly increased from about 0.30 to 0.55 V after 4 h. The low initial OCP, can be explained by a higher activity of H_2_, generated by the H_3_PO_3_ catalytic oxidation (see eq 1). The H_2_ generated at the Pt surface forms a redox couple with H^+^ (i.e., the gas diffusion electrode behaved partly as a reversible hydrogen electrode). However, as the electrolyte solution was bubbled with N_2_ during the measurement, the generated H_2_ was continuously stripped from the electrode resulting in a low H_2_ activity near the electrode surface. The increase in time of the OCP can then be attributed to a steady deactivation/poisoning of the Pt surface, most likely by the formation and adsorption of intermediate oxidation products, which slows down the catalytic oxidation of H_3_PO_3_. A similar deactivation of a Pt electrode during anodic oxidation of H_3_PO_3_ has already been observed in our previous work.^15^ Congenitally, the overall rate of the H_3_PO_3_ oxidation will depend on the catalytic activity of the accessible Pt surface and the intensity of H_3_PO_3_ transport toward the Pt surface in the gas diffusion electrode. In contrast to Pt/C dispersed in the electrolyte solution, the gas diffusion electrode represents a compact porous system with significantly constrained mass transport. In addition to that, the solution volume to the Pt/C mass ratio in the experiment with the GDE was more than 35 times higher compared to the Pt/C suspension in the electrolyte solution. These are the likely reasons why the bulk concentration of H_3_PO_3_ did not change significantly during the 4 h experiment in the electrochemical cell.

**Figure 2:**
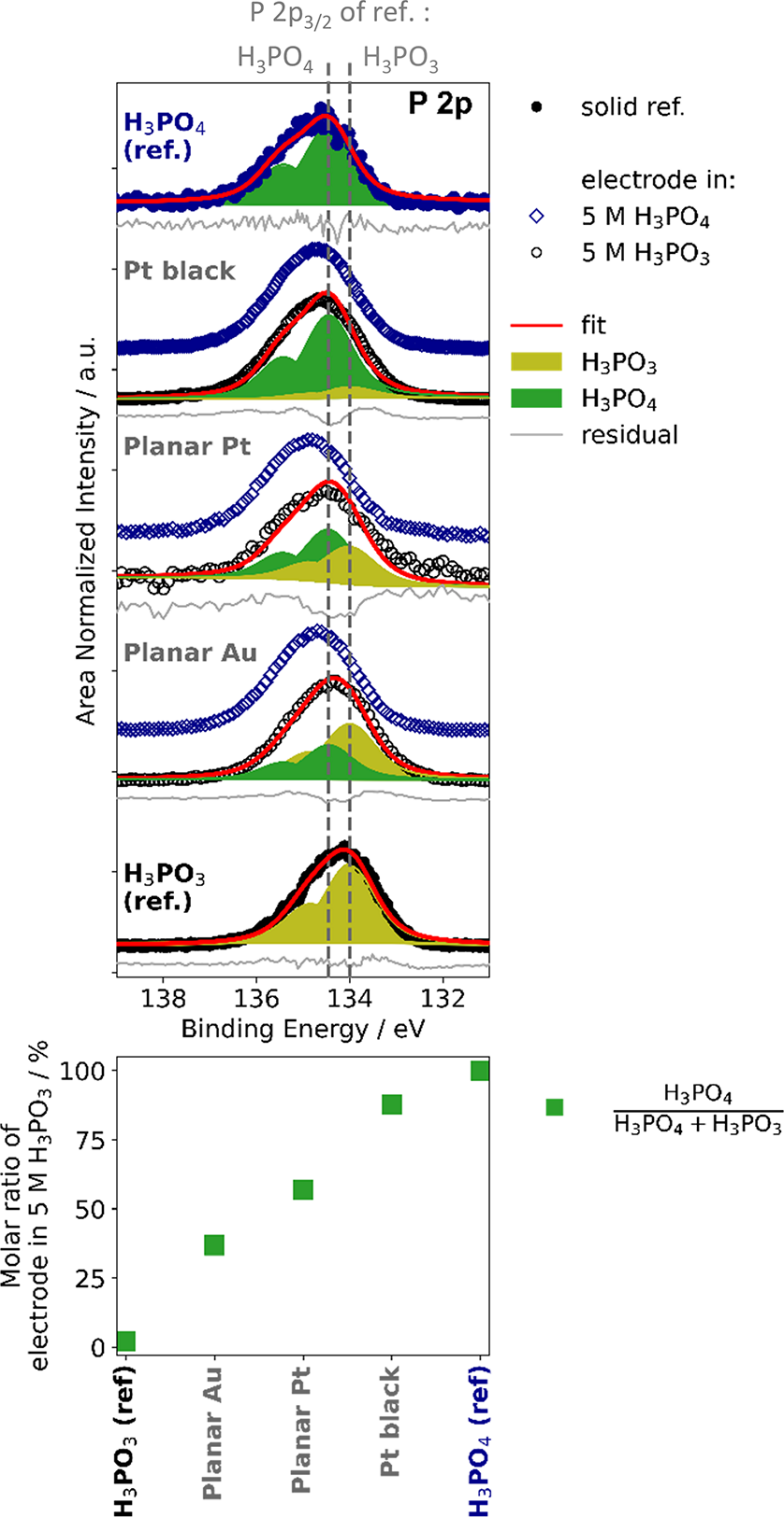
Top: P 2p XPS core level spectra of Au and Pt electrodes previously immersed in either 5 mol dm^–3^ (5M) H_3_PO_3_ or H_3_PO_4_ solutions after subsequent drying (“H_3_PO_3_-treated electrode” or “H_3_PO_4_-treated electrode”). For comparison, the P 2p spectra of solid H_3_PO_3_ and H_3_PO_4_ samples are shown as references. The vertical dashed lines indicate the position of the P 2p_3/2_ binding energy of each solid H_3_PO_3_ and H_3_PO_4_ reference. Similar spectral shapes and binding energy positions are observed for different electrodes previously treated in 5 mol of dm^–3^ H_3_PO_4_, but electrodes previously treated in 5 mol of dm^–3^ H_3_PO_3_ each possess varying spectral shapes and binding energy positions. Bottom: Molar ratio of the H_3_PO_4_ : (H_3_PO_3_ + H_3_PO_4_), hereafter will be called “H_3_PO_4_ molar ratio”, related contributions to the P 2p line determined for the different H_3_PO_3_ treated electrodes along with the references.

The same phenomenon (i.e., oxidation of H_3_PO_3_ to H_3_PO_4_ on a Pt surface) was also investigated by XPS. In this experiment, Pt and, as a reference, Au electrodes were immersed in 5 mol dm^–3^ H_3_PO_3_ solution in an inert atmosphere with subsequent freeze-drying in UHV prior to the XPS measurement (hereafter will be referred to as “H_3_PO_3_-treated electrode”). For comparison, a similar measurement was made with a similar set of electrodes immersed into 5 mol dm^–3^ H_3_PO_4_, hereafter will be referred to as “H_3_PO_4_-treated electrode”. Figure 2 shows the respective P 2p spectra of these samples along with solid H_3_PO_3_ and solid H_3_PO_4_ references (i.e., reference of both acids at the pristine state).

For all of the H_3_PO_4_-treated metal electrodes (planar Au, planar Pt, and Pt black), the P 2p spectra look very similar, resembling that of the solid H_3_PO_4_ reference–corroborated by detailed peak fitting analysis (see Figure S3 in the SI), indicating that H_3_PO_4_ is stable on those electrodes. For the H_3_PO_3_-treated electrodes, we find more complex P 2p spectra changing in the spectral shape and binding energy position for the different electrodes. The fit analysis of the P 2p line of the planar Pt electrode reveals two contributions. The low binding energy P 2p component agrees with that of the solid H_3_PO_3_ reference, and the high binding energy component agrees with that of the solid H_3_PO_4_ reference. For the planar Pt electrode, it was found that the adsorbed H_3_PO_4_ and H_3_PO_3_-like species are present in a ratio of 63.0/37.0, as shown in the bottom panel of Figure 2 (the detailed value of H_3_PO_4_: (H_3_PO_3_ + H_3_PO_4_) molar ratio determination, hereafter will be called “H_3_PO_4_ molar ratio”, can be found in Table S2 and S3 in the SI, for the solid references and the acid-treated electrode, respectively). This implies that a substantial part of H_3_PO_3_ has been oxidized to H_3_PO_4_ upon adsorption on the planar Pt electrode. Remarkably, when the same experiment was performed with a planar Au electrode, the XPS analysis revealed that only 44.0% of the H_3_PO_3_ was oxidized to H_3_PO_4_. These results agree with the above suggestion that Pt catalyzes the oxidation of H_3_PO_3_ to H_3_PO_4_. Oxidation also occurred on planar Au, although to a lesser extent compared to planar Pt. Additionally, an even higher content of H_3_PO_4_ compared to the planar Pt was observed in the XPS measurement performed using high surface area Pt black (16.0% H_3_PO_3_ and 84.0% H_3_PO_4_). This might indicate a higher extent of H_3_PO_3_ oxidation either due to the larger effective surface area of Pt in contact with H_3_PO_3_ (as shown in Figure S2 in the SI) or due to the nature of the active sites on the Pt black (for the adsorption and oxidation of H_3_PO_3_). Furthermore, additional electrochemical characterization of open circuit potential (OCP) monitoring was conducted using three different types of electrodes (Pt black, planar Pt, and planar Au) in a 5 mol dm^–3^ H_3_PO_3_ electrolyte. The results are depicted in Figure S4, demonstrating a gradual increase in OCP over time for all the electrodes (∼ +0.45 V to +0.61 V vs RHE for both Pt electrodes, ∼ +0.51 to 0.61 V vs RHE for planar Au), likely related to the oxidation of H_3_PO_3_ and subsequent blockage of Pt catalysts by the products of H_3_PO_3_ oxidation, as previously observed in the OCP measurements of aqueous H_3_PO_3_ electrolyte with GDE (Figure 1E). Each electrode exhibits a distinct rate of OCP increase over time, with Pt black demonstrating the highest rate followed by the planar Pt electrode and the planar Au electrode. Additionally, the OCP of planar Au only changes slightly during the experiments compared to the other electrodes. The difference in the OCP change rates agrees with the XPS-derived H_3_PO_4_ molar ratio of the H_3_PO_3_-treated electrode shown in Figure 2, with a higher concentration of converted H_3_PO_4_ observed for the Pt black than for the planar Pt and planar Au electrode. However, the exact underlying nature of the electrode surface blocking remains unclear.

**Figure 3:**
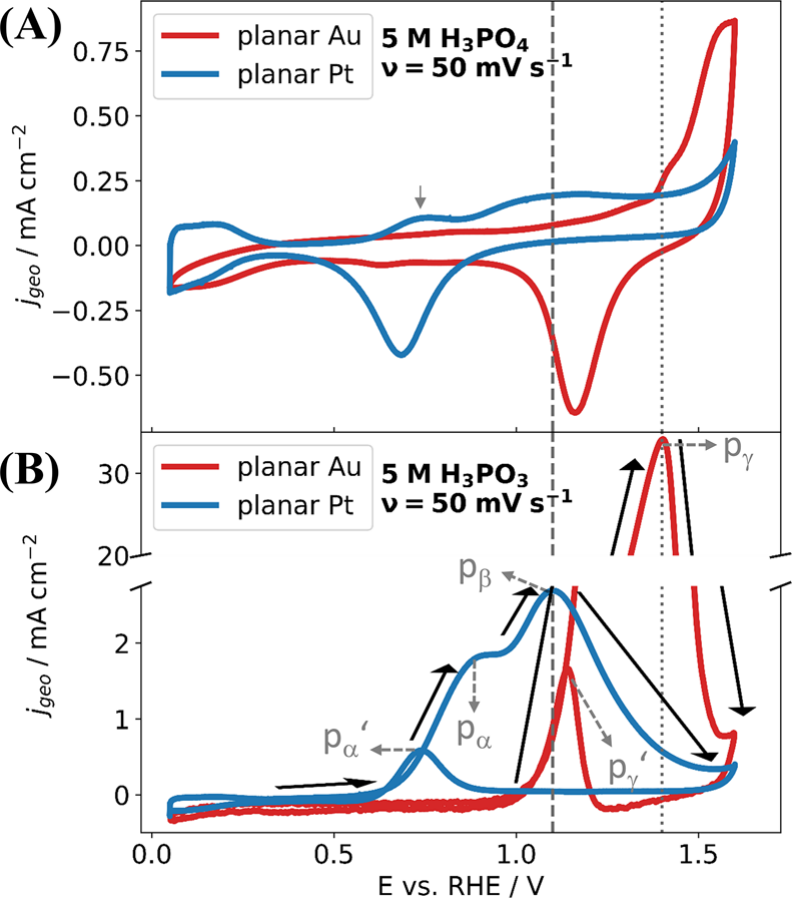
Cyclic voltammogram (CV) of planar Pt and Au electrodes in (A) 5 mol dm^–3^ (5 M) H_3_PO_4_ and (B) in 5 mol dm^–3^ (5 M) H_3_PO_3_, using the scan rate of 50 mV s^–1^. The direction of the current response during the positive-going potential sweep in panel (B) is shown with a black arrow. Gray dashed line and gray dotted line correspond to the peak potentials of H_3_PO_3_ oxidation on surface oxides, for planar Pt and planar Au electrode in panel (B), respectively. Lower H_3_PO_3_ onset anodic oxidation potential observed in CV recorded planar Pt electrode-5 mol dm^–3^ H_3_PO_3_ (pα), compared to planar Au electrode-5 mol dm^–3^ H_3_PO_3_ (pγ).

For further insight into the different oxidation behavior of H_3_PO_3_ on these electrodes, electrochemical characterization, i.e., cyclic voltammograms of planar Pt and Au electrodes were recorded in 5 mol dm^–3^ (5 M) H_3_PO_3_ and H_3_PO_4_ electrolyte solutions, as illustrated in Figure 3.

The voltammograms recorded in H_3_PO_4_ contain features typically observed for Pt and Au electrodes in acidic electrolytes (e.g., formation of oxide layer on the metal electrodes during the positive-going potential sweep, as well as the subsequent reduction of the formed oxide in the negative-going potential sweep), as shown in Figure 3A. The oxidation peak at +0.75 V present in the voltammogram of Pt in 5 mol dm^–3^ of H_3_PO_4_ (indicated by an arrow in Figure 3A) suggests possible minor contamination of the system by H_3_PO_3_. At the negative-going potential sweep, a peak corresponding to the reduction of H_3_PO_4_ to H_3_PO_3_ was not observed, since the equilibrium potential for this reaction lies beyond the lower potential limit of the recorded voltammogram (see eq 2). The voltammograms recorded in H_3_PO_3_ solutions, as illustrated Figure 3B, showed different behavior: the voltammograms measured on Pt reveal a rather complex situation, where the presence of an oxidation feature pα (at potentials above +0.5 V) is attributed to the anodic oxidation of H_3_PO_3_ to H_3_PO_4_ via H_2_O on metallic Pt surface^14^ and pβ is ascribed to the oxidation of H_3_PO_3_ to H_3_PO_4_ on the weakly oxidized Pt surface.^14^ On the negative-going potential sweep, peak pα′ is presumably related to the secondary oxidation of H_3_PO_3_, which occurred following the reduction of PtO sites (resulting in active Pt sites), as suggested in the previous study.^14^ Additional measurements further support the findings of ref (14), revealing that pα indeed corresponds to the oxidation of H_3_PO_3_ to H_3_PO_4_, presented in section S8 of the SI and Figure S6. For the anodic oxidation peak pα (Figure 3B), the peak current density of about 2 mA cm^–2^ is rather low considering the high concentration of H_3_PO_3_. On the other hand, the voltammogram of H_3_PO_3_ on the Au electrode only showed one peak feature (pγ) in the positive-going potential sweep, likely corresponding to anodic oxidation of H_3_PO_3_ to H_3_PO_4_, as well as peak pγ′ in the negative-going potential sweep, possibly indicating secondary oxidation of H_3_PO_3_ after the reduction of oxide layer on the metallic, i.e., oxide free Au surface. The anodic H_3_PO_3_ oxidation on Au did not start below +1.0 V and oxidation peak current densities as high as 30 mA cm^–2^ were observed.

A comparison of voltammograms reveals that the H_3_PO_3_ onset oxidation potential on Pt is around 400 mV lower than that on Au, confirming a significantly higher activity of Pt toward H_3_PO_3_ oxidation at electrode potentials below +1.0 V vs RHE, i.e., before the Pt surface is oxidized. Moreover, the higher activity of planar Pt compared to planar Au is in line with the significant decrease of XPS-derived molar ratio of H_3_PO_3_ on planar Pt compared to planar Au shown in Figure 2. However, the low oxidation current density at Pt suggests that the Pt surface is easily blocked/poisoned by adsorbed H_3_PO_3_ or its oxidation products, which is in agreement with the increasing OCP value over time in the previously discussed electrochemical experiment with the GDE, in Figure 1E and Figure S4 as well as previous observation.^14,15^ Furthermore, for both planar metal electrodes, the oxidation of H_3_PO_3_ stops once the metallic surface is covered by higher oxides (shown by gray dashed lines in Figure 3), indicating that metallic presence is favorable toward the oxidation of H_3_PO_3_. Previous investigations have also suggested that H_3_PO_3_ adsorb on top of Pt within the potential range of 0.0 V to +0.5 V vs RHE, where metallic Pt is accessible from the electrolyte phase.^17^ This adsorption occurs in its pyramidal form through a P–Pt interaction. The presence of pyramidal tautomer on Pt might explain the strong oxidation of H_3_PO_3_ on Pt. Since the pyramidal H_3_PO_3_ tautomer is highly reactive, it is more susceptible to react with H_2_O, and thus the H_3_PO_3_ may undergo oxidation to H_3_PO_4_ through H_2_O, via eq 1. Moreover, it is likely that the adsorption also takes place even at potentials between +0.5 and +1.0 V, although in this potential window, the amount of adsorbed H_3_PO_3_ decreases due to its oxidation. The adsorption behavior of H_3_PO_3_ on the oxidized Pt surface (e.g., > + 1.2 V) remains, however, unclear. Yet, the decreasing current density indicates that either the highly oxidized Pt surface is unable to oxidize H_3_PO_3_ or the surface gets blocked by the adsorbed products. Analogous information can be drawn from the voltammogram of a planar Au electrode presented in Figure 3B. Further systematic spectro-electrochemical investigations during potential bias application are necessary to gain detailed insights into the Pt|aq. H_3_PO_3_ interface processes under these conditions; however, these are beyond the scope of this study and will be explored prospectively in the future.

To determine if H_3_PO_3_ oxidation observed with XPS could also be observed in an environment close to ambient pressure, and for the possibility of electrochemical characterization under the same condition with the performed XPS, *in situ* AP-HAXPES combined with the “dip-and-pull” method was performed on the Pt|H_3_PO_3_ interface.

**Figure 4:**
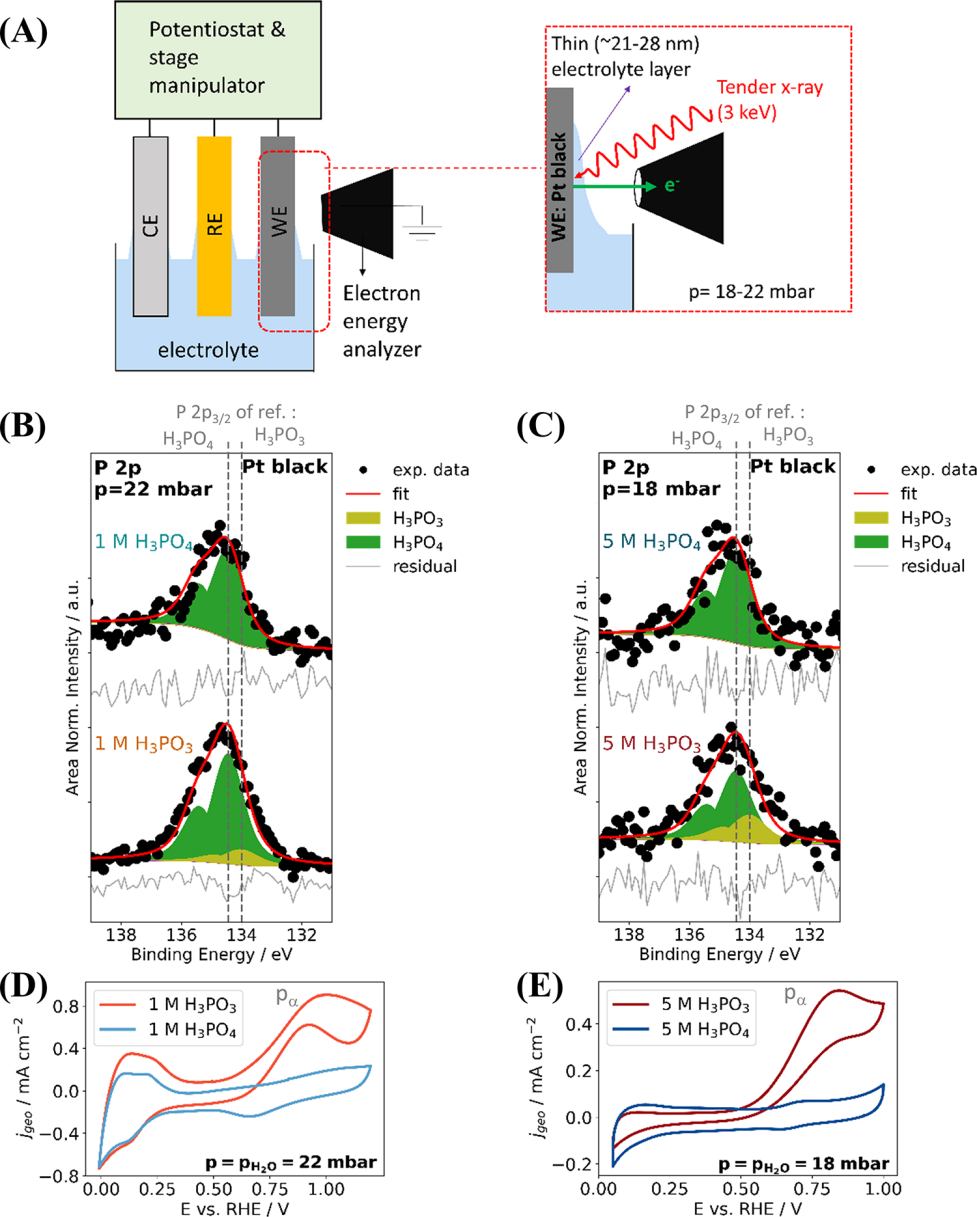
(A) Illustration of the *in situ* AP-HAXPES combined with “dip-and-pull” experimental configuration. *In situ* AP-HAXPES P 2p core level spectra of a Pt black electrode in (B) 1 mol dm^–3^ (1 M) and (C) 5 mol dm^–3^ (5 M) aqueous (aq.) acids (H_3_PO_3_ and H_3_PO_4_). All AP-HAXPES measurements were conducted in an open circuit potential (OCP). The average OCP of the aq. H_3_PO_3_ is +0.47 V and +0.4 V, for the 1 M and the 5 M electrolytes, respectively. For the aq. H_3_PO_4_, the average OCP is +0.74 and +0.67 V, for the 1 and the 5 M electrolytes, respectively. The binding energy in the AP-HAXPES measurements was calibrated to the observed OCP. Cyclic voltammograms (CV) of the Pt black electrode were recorded in the configuration detailed in panel (A) with (D) 1 mol dm^–3^ (1 M) and (E) 5 mol dm^–3^ (5 M) aqueous acids. Each CV was recorded at a scan rate of 50 mV s^–1^. Deconvolution of *in situ* AP-HAXPES spectra recorded in 1 mol dm^–3^ (1 M) and 5 mol dm^–3^ (5 M) H_3_PO_3_ show a prevalent contribution of H_3_PO_4_ and a small contribution of H_3_PO_3_, even though CVs recorded under the same condition show a strong characteristic oxidation peak of H_3_PO_3_.

Figure 4A shows an illustration of the *in situ* “dip-and-pull” experimental configuration. A thin electrolyte layer is formed when the Pt black working electrode is dipped into the electrolyte and then carefully pulled out (thus the terminology “dip-and-pull”). The resulting electrode|thin electrolyte layer interface was probed by AP-HAXPES for 1 mol of dm^–3^ and 5 mol of dm^–3^ solutions of H_3_PO_3_ and H_3_PO_4_. Representative O 1s and Pt 4f core level spectra are shown in Figure S7 in the SI, demonstrating that the probed regions are indeed electrode|thin layer electrolyte interfaces. As an experimental validation that the probed electrode|thin electrolyte layer interface was connected to the bulk electrolyte solution, the corresponding O 1s and Pt 4f core level under bias potential was made and shown in Figure S8, in section S12 of the SI.

The *in situ* AP-HAXPES P 2p core level spectra of the electrolyte layer interface and cyclic voltammograms conducted in the “dip-and-pull” measurement configuration are shown in Figure 4B,C and Figure 4D,E, respectively. Despite the limited signal-to-noise ratio of the AP-HAXPES data, it is clear that the H_3_PO_4_ contribution dominates the spectra independent of the used electrolyte and/or concentration, corroborating the oxidation of H_3_PO_3_ to H_3_PO_4_ occurring at the OCP, as discussed in the previous section. The P 2p peak deconvolution confirms that a large contribution of the spectrum of the Pt|thin H_3_PO_3_ electrolyte interface in both 1 and 5 mol of dm^–3^ was arising from H_3_PO_4_, as illustrated in Figure 4B,C (detail of the fitting parameters given in Table S7 in the SI). In the 1 mol dm^–3^ solution, a higher contribution of H_3_PO_4_ molar ratio (87.0%) was observed compared to (70.0% H_3_PO_4_) in the 5 mol dm^–3^ solution. This difference is in line with the possibility of H_3_PO_3_ oxidation by H_2_O (as discussed previously and summarized by eq 1), due to the higher H_2_O:H_3_PO_3_ molar ratio in 1 mol dm^–3^ compared to 5 mol dm^–3^ H_3_PO_3_ solution. Furthermore, the averaged OCP values for Pt|1 mol dm^–3^ H_3_PO_3_ and Pt|5 mol dm^–3^ H_3_PO_3_ in this study are +0.47 and +0.41 V vs RHE, respectively, which are similar to the potential at which the majority of adsorbed species on Pt correspond to H_3_PO_3_ in its pyramidal form (at +0.4 V), as reported by Gomes et al.^17^ However, according to our findings, it seems that the notion of Pt “equilibrium surface coverage” by H_3_PO_3_ previously reported by Gomes et al.^17^ has not been correctly attributed. The oxidation of H_3_PO_3_ to H_3_PO_4_ on Pt, likely through the reaction of highly reactive pyramidal H_3_PO_3_ with H_2_O via eq 1, as previously discussed, provides a new interpretation of the H_3_PO_3_ adsorption on Pt: the equilibrium coverages are to be reconsidered as steady-state ones, i.e., following the oxidation of H_3_PO_3_ on Pt.

In addition, the XPS analysis on the Pt 4f core level, revealing the sole presence of metallic Pt (Figure S6B), supports the aforementioned interpretation. It should be noted that since the detection limit of the “dip-and-pull” AP-HAXPES under the experimental condition is around a monolayer, it is plausible that a very small amount (e.g., a monolayer or less) of a surface “oxide” might exist on the Pt surface (i.e., PtO_*x*_). If a PtO_*x*_ monolayer presents on the electrode surface, it could oxidize a portion of H_3_PO_3_^16^ (e.g., through the reaction: PtO + H_3_PO_3_ ⇆ Pt + H_3_PO_4_); thus, their impact on the oxidation of H_3_PO_3_ cannot be ruled out. Yet, even with the assumption of a full PtO_*x*_ monolayer coverage on the electrode surface, this factor alone would not be able to explain the high H_3_PO_4_ molar ratio observed in the AP-HAXPES experiment. The H_3_PO_4_ molar ratio observed in this experiment is greater than the estimated H_3_PO_4_ molar ratio generated solely due to oxidation by the PtO_*x*_ monolayer. Details for theoretical H_3_PO_4_ molar ratio estimation, which arise solely due to the presence of a PtO_*x*_ monolayer, can be found in section S13 of the SI, Table S8. This suggests that other processes mainly take place in the oxidation of the H_3_PO_3_ and provides another indication that H_3_PO_3_ is mainly oxidized by H_2_O on metallic Pt surface as previously discussed in the context of CV of Pt|5 mol dm^–3^ H_3_PO_3_.

Interestingly, even though AP-HAXPES shows a prevalent H_3_PO_4_ contribution in both 1 and 5 mol dm^–3^ H_3_PO_3_, cyclic voltammogram recorded under the same condition (Figure 4D,E) shows strong similarities to voltammogram made with pure H_3_PO_3_ electrolyte rather than pure H_3_PO_4_ electrolyte (CVs are shown in Figure S5 in the SI). In particular, characteristic oxidation peaks corresponding to H_3_PO_3_ oxidation are visible in the voltammograms measured in H_3_PO_3_ electrolyte,^14^ while voltammograms obtained in H_3_PO_4_ solution possess mainly features that are expected for Pt electrode under polarization in an acidic environment. To resolve the contradicting information provided by the electrochemical and AP-HAXPES experiments, a closer look needs to be taken into the measurement configuration of the *in situ* “dip-and-pull” experimental setup.

As depicted in Figure 4A, considering the given measurement configuration, AP-HAXPES probes the working electrode|thin electrolyte layer interface with electrolyte thicknesses between 21 and 28 nm (for the data shown in Figure 4B,C), which is similar to previously reported liquid meniscus thickness values.^23,34−38^ Details for the estimation of the electrolyte layer thickness for these experiments are given in section S10 of the SI, Table S6. At this Pt electrode|thin H_3_PO_3_ electrolyte interface, the catalytic oxidation of H_3_PO_3_ to H_3_PO_4_ by H_2_O occurs according to eq 1. However, since the AP-HAXPES analysis of the electrode|electrolyte interface is in this case limited to a region where the electrolyte is very thin, there is a significant increase of the “local” H_3_PO_4_ concentration in this meniscus region due to the limited mass transport between the ∼21–28 nm thick electrolyte layer and electrolyte solution bulk. The mass and charge transport limitation in the thin layer meniscus used for the “dip-and-pull” experiments has also been documented in other studies.^37,39^ Additional *in situ* “dip-and-pull” AP-HAXPES experiments were performed at the Pt|aq. H_3_PO_3_ interface with a varying electrolyte layer thickness (see section S14 of the SI and the discussion in conjunction with Figures S10 and S11 for more details) shows the reproducibility of the measurements and indicates rather stable conditions even beyond the meniscus region with extremely thin electrolyte thickness. In contrast, voltammetry results not only arise from this small electrode|electrolyte region but are dominated by a much larger region where the electrode is surrounded by the electrolyte bulk solution (i.e., part of the electrode immersed in the solution), ensuring intensive H_3_PO_3_ supply also to the electrode|electrolyte interface. Therefore, CV measurements probing all regions of the electrode in contact with the electrolyte indicate the anodic oxidation of H_3_PO_3_. Furthermore, for confirmation that the voltammogram recorded in this experiment is indeed sensitive to the state of H_3_PO_3_ in the bulk solution, an additional electrochemical characterization with a specially tailored Pt black electrode in a fully immersed electrode configuration as well as thin film-electrolyte-only configuration was made and presented in Figure S12 in section S15 of the SI. In summary, with the *in situ* AP-HAXPES, the information emerges from the probed Pt|H_3_PO_3_ thin layer interface in the upper part of the meniscus where H_3_PO_3_ was mostly oxidized, while in contrast, voltammetry response substantially arises from the H_3_PO_3_ oxidation outside of the meniscus (i.e., immersed region), where the formed H_3_PO_4_ is constantly replaced by the fresh electrolyte in the bulk.

## 4 Conclusions

The stability of H_3_PO_3_ in aqueous solutions at ambient temperature in the absence of O_2_ has been investigated. Despite the inherent thermodynamic H_3_PO_3_ instability, the solution, on its own, is stable. This can be attributed to the fact that in ambient conditions, H_3_PO_3_ exists practically completely in its significantly more stable and less reactive tetrahedral tautomeric form. When the solution is brought into direct contact with a Pt surface, H_3_PO_3_ adsorbs at the Pt surface most likely in the highly reactive pyramidal tautomeric form and undergoes chemical oxidation by H_2_O to H_3_PO_4_ accompanied by H_2_ generation. This oxidation to H_3_PO_4_ was confirmed by IEC, *ex-situ* XPS on H_3_PO_3_-treated electrodes, and *in situ* “dip-and-pull” AP-HAXPES experiments on Pt|aqueous H_3_PO_3_ interfaces. Therefore, it is important to consider this behavior when investigating solutions and especially interfaces where H_3_PO_3_ is present. An example of such a system is the electrified Pt|H_3_PO_4_ interface in HT-PEMFCs where H_3_PO_3_ was indicated to form. Finally, the results of this work significantly change the interpretation of the “equilibrium surface coverages of Pt by H_3_PO_3_” and adsorption isotherms compared with the interpretation presented in the previous works. In light of the results presented in this work, these “equilibrium coverages” are actually to be seen as steady-state ones. To bridge the gap of the behavior of H_3_PO_3_/H_3_PO_4_ in real-world HT-PEMFC operation conditions, similar experiments at increased H_3_PO_4_ concentration, elevated temperatures, and under potential bias to the Pt electrodes are required.

## Supporting Information Available

The Supporting Information contains: Topography and surface roughness determination of planar Au and planar Pt electrodes; Pt black electrode morphology and electrodeposition profile; P 2p core level measurements for planar Au, planar Pt, and Pt black electrodes in H_3_PO_3_ and H_3_PO_4_; Fitting parameters for the AP-HAXPES on solid crystalline H_3_PO_3_ and H_3_PO_4_ acids; Fitting parameters for the XPS on the acid-treated electrodes; Open circuit potential (OCP) monitoring of 5 mol dm^–3^ H_3_PO_3_ in contact with planar Pt, planar Au, and Pt black electrodes; Cyclic voltammograms of 5 mol dm^–3^ H_3_PO_3_ and 5 mol dm^–3^ H_3_PO_4_ solutions with different upper limit potential; CV of 10 mmol dm^–3^ H_3_PO_3_ + 0.5 mmol dm^–3^ H_3_PO_4_ using a Pt electrode; Validation of the observations at the electrode|electrolyte interface; Determination of electrolyte layer thickness on the electrode surface for the in situ AP-HAXPES coupled with the “Dip-and-pull” method; Fitting parameters and quantification for the P 2p core level in the *in situ* AP-HAXPES coupled with “Dip-and-pull” method; Validation of continuous thin film electrolyte from the probed Pt|thin electrolyte interface to the bulk electrolyte; Estimation of H_3_PO_4_ molar fraction resulting from the oxidation of H_3_PO_3_ by a monolayer of PtO_*x*_; *In situ* “Dip-and-pull” AP-HAXPES measurements of the Pt-electrode|aqueous H_3_PO_3_ electrolyte interface; Comparison of the electrochemical characterization conducted in thin-film only configuration versus fully immersed electrode configuration (PDF)

## References

[ref1] Chandan A.; Hattenberger M.; El-kharouf A.; Du S.; Dhir A.; Self V.; Pollet B. G.; Ingram A.; Bujalski W. High Temperature (HT) Polymer Electrolyte Membrane Fuel Cells (PEMFC) – A Review. J. Power Sources 2013, 231, 264–278. 10.1016/j.jpowsour.2012.11.126.

[ref2] Araya S. S.; Zhou F.; Liso V.; Sahlin S. L.; Vang J. R.; Thomas S.; Gao X.; Jeppesen C.; Kær S. K. A Comprehensive Review of PBI-Based High Temperature PEM Fuel Cells. Int. J. Hydrogen Energy 2016, 41 (46), 21310–21344. 10.1016/j.ijhydene.2016.09.024.

[ref3] Li Q.; He R.; Gao J.-A.; Jensen J. O.; Bjerrum Niels. J. The CO Poisoning Effect in PEMFCs Operational at Temperatures up to 200 °C. J. Electrochem. Soc. 2003, 150 (12), A1599 10.1149/1.1619984.

[ref4] Li Q.; He R.; Jensen J. O.; Bjerrum N. J. Approaches and Recent Development of Polymer Electrolyte Membranes for Fuel Cells Operating above 100 °C. Chem. Mater. 2003, 15 (26), 4896–4915. 10.1021/cm0310519.

[ref5] Pan C.; He R.; Li Q.; Jensen J. O.; Bjerrum N. J.; Hjulmand H. A.; Jensen A. B. Integration of High Temperature PEM Fuel Cells with a Methanol Reformer. J. Power Sources 2005, 145 (2), 392–398. 10.1016/j.jpowsour.2005.02.056.

[ref6] Qi A.; Peppley B.; Karan K. Integrated Fuel Processors for Fuel Cell Application: A Review. FPT 2007, 88 (1), 3–22. 10.1016/j.fuproc.2006.05.007.

[ref7] Weng F.; Cheng C.-K.; Chen K.-C. Hydrogen Production of Two-Stage Temperature Steam Reformer Integrated with PBI Membrane Fuel Cells to Optimize Thermal Management. Int. J. Hydrogen Energy 2013, 38 (14), 6059–6064. 10.1016/j.ijhydene.2013.01.090.

[ref8] Avgouropoulos G.; Paxinou A.; Neophytides S. In Situ Hydrogen Utilization in an Internal Reforming Methanol Fuel Cell. Int. J. Hydrogen Energy 2014, 39 (31), 18103–18108. 10.1016/j.ijhydene.2014.03.101.

[ref9] Nart F. C.; Iwasita T. On the Adsorption of H_2_PO_4^–^_ and H_3_PO_4_ on Platinum: An in Situ FT-Ir Study. Electrochim. Acta 1992, 37 (3), 385–391. 10.1016/0013-4686(92)87026-V.

[ref10] Kaserer S.; Caldwell K. M.; Ramaker D. E.; Roth C. Analyzing the Influence of H_3_PO_4_ as Catalyst Poison in High Temperature PEM Fuel Cells Using *in-Operando* X-Ray Absorption Spectroscopy. J. Phys. Chem. C 2013, 117 (12), 6210–6217. 10.1021/jp311924q.

[ref11] Vogel W. M.; Baris J. M. Changes in the Surface of Platinum in Hot Concentrated Phosphoric Acid at Low Potentials. Electrochim. Acta 1978, 23 (5), 463–466. 10.1016/0013-4686(78)87047-9.

[ref12] Sugishima N.; Hinatsu J. T.; Foulkes F. R. Phosphorous acid Impurities in Phosphoric Acid Fuel Cell Electrolytes: II. Effects on the Oxygen Reduction Reaction at Platinum Electrodes. J. Electrochem. Soc. 1994, 141 (12), 3332 10.1149/1.2059335.

[ref13] Doh W. H.; Gregoratti L.; Amati M.; Zafeiratos S.; Law Y. T.; Neophytides S. G.; Orfanidi A.; Kiskinova M.; Savinova E. R. Scanning Photoelectron Microscopy Study of the Pt/Phosphoric-Acid-Imbibed Membrane Interface under Polarization. ChemElectroChem 2014, 1 (1), 180–186. 10.1002/celc.201300134.

[ref14] Prokop M.; Bystron T.; Bouzek K. Electrochemistry of Phosphorous and Hypophosphorous Acid on a Pt Electrode. Electrochim. Acta 2015, 160, 214–218. 10.1016/j.electacta.2015.01.097.

[ref15] Prokop M.; Bystron T.; Paidar M.; Bouzek K. H_3_PO^_3_^ Electrochemical Behaviour on a Bulk Pt Electrode: Adsorption and Oxidation Kinetics. Electrochim. Acta 2016, 212, 465–472. 10.1016/j.electacta.2016.07.045.

[ref16] Gomes B. F.; Prokop M.; Bystron T.; Loukrakpam R.; Lobo C. M. S.; Kutter M.; Günther T. E.; Fink M.; Bouzek K.; Roth C. Effect of Phosphoric Acid Purity on the Electrochemically Active Surface Area of Pt-Based Electrodes. J. Electroanal. Chem. 2022, 918, 116450 10.1016/j.jelechem.2022.116450.

[ref17] Gomes B. F.; Prokop M.; Bystron T.; Loukrakpam R.; Melke J.; Lobo C. M. S.; Fink M.; Zhu M.; Voloshina E.; Kutter M.; Hoffmann H.; Yusenko K. V.; Buzanich A. G.; Röder B.; Bouzek K.; Paulus B.; Roth C. Following Adsorbed Intermediates on a Platinum Gas Diffusion Electrode in H_3_PO_3_ -Containing Electrolytes Using In Situ X-Ray Absorption Spectroscopy. ACS Catal. 2022, 12 (18), 11472–11484. 10.1021/acscatal.2c02630.

[ref18] He Q.; Yang X.; Chen W.; Mukerjee S.; Koel B.; Chen S. Influence of Phosphate Anion Adsorption on the Kinetics of Oxygen Electroreduction on Low Index Pt(Hkl) Single Crystals. Phys. Chem. Chem. Phys. 2010, 12 (39), 12544 10.1039/c0cp00433b. 20725683

[ref19] Deng Y.-J.; Wiberg G. K. H.; Zana A.; Arenz M. On the Oxygen Reduction Reaction in Phosphoric Acid Electrolyte: Evidence of Significantly Increased Inhibition at Steady State Conditions. Electrochim. Acta 2016, 204, 78–83. 10.1016/j.electacta.2016.04.065.

[ref20] Moulder J. F.; Stickle W. F.; Sobol P. E.; Bomben K. D. Handbook of X-Ray Photoelectron Spectroscopy: A Reference Book of Standard Spectra for Identification and Interpretation of XPS Data; Physical Electronics, Incorporation, Eds.; Physical Electronics: Eden Prairie, Minn., 1995.

[ref21] Favaro M.; Clark P. C. J.; Sear M. J.; Johansson M.; Maehl S.; van de Krol R.; Starr D. E. Spectroscopic Analysis with Tender X-Rays: SpAnTeX, a New AP-HAXPES End-Station at BESSY II. Surf. Sci. 2021, 713, 121903 10.1016/j.susc.2021.121903.

[ref22] Schaefers F.; Mertin M.; Gorgoi M. KMC-1: A High Resolution and High Flux Soft x-Ray Beamline at BESSY. Rev. Sci. Instrum. 2007, 78 (12), 123102 10.1063/1.2808334. 18163715

[ref23] Axnanda S.; Crumlin E. J.; Mao B.; Rani S.; Chang R.; Karlsson P. G.; Edwards M. O. M.; Lundqvist M.; Moberg R.; Ross P.; Hussain Z.; Liu Z. Using “Tender” X-Ray Ambient Pressure X-Ray Photoelectron Spectroscopy as A Direct Probe of Solid-Liquid Interface. Sci. Rep. 2015, 5 (1), 9788 10.1038/srep09788. 25950241 PMC4650780

[ref24] Favaro M.; Abdi F.; Crumlin E.; Liu Z.; van de Krol R.; Starr D. Interface Science Using Ambient Pressure Hard X-Ray Photoelectron Spectroscopy. Surfaces 2019, 2 (1), 78–99. 10.3390/surfaces2010008.

[ref25] Favaro M. Stochastic Analysis of Electron Transfer and Mass Transport in Confined Solid/Liquid Interfaces. Surfaces 2020, 3 (3), 392–407. 10.3390/surfaces3030029.

[ref26] Salmeron M.; Schlogl R. Ambient Pressure Photoelectron Spectroscopy: A New Tool for Surface Science and Nanotechnology. Surf. Sci. Rep. 2008, 63 (4), 169–199. 10.1016/j.surfrep.2008.01.001.

[ref27] de Groot F.; Kotani A. Core Level Spectroscopy of Solids; 0 ed.; CRC Press, 2008. 10.1201/9781420008425.

[ref28] Piacentini M.; Khumalo F. S.; Olson C. G.; Anderegg J. W.; Lynch D. W. Optical Transitions, XPS, Electronic States in NiPS3. Chem. Phys. 1982, 65 (3), 289–304. 10.1016/0301-0104(82)85205-1.

[ref29] Poirier D. M.; Weaver J. H. InP(110) by XPS. Surf. Sci. Spectra 1993, 2 (3), 256–262. 10.1116/1.1247707.

[ref30] Dudzik E.; Müller C.; McGovern I. T.; Lloyd D. R.; Patchett A.; Zahn D. R. T.; Johal T.; McGrath R. H2S Adsorption on the (110) Surfaces of III–V Semiconductors. Surf. Sci. 1995, 344 (1–2), 1–10. 10.1016/0039-6028(95)00799-7.

[ref31] Rosenberg R. A.; LaRoe P. R.; Rehn V.; Loubriel G. M.; Thornton G. Soft-x-Ray Photoelectron-Yield Spectrum of InP(110) from 65 to 195 EV. Phys. Rev. B 1983, 28 (10), 6083–6085. 10.1103/PhysRevB.28.6083.

[ref32] Newville M.; Stensitzki T.; Allen D. B.; Ingargiola A. LMFIT: Non-Linear Least-Square Minimization and Curve-Fitting for Python; Zenodo, 2014. 10.5281/ZENODO.11813.

[ref34] Ali-Löytty H.; Louie M. W.; Singh M. R.; Li L.; Sanchez Casalongue H. G.; Ogasawara H.; Crumlin E. J.; Liu Z.; Bell A. T.; Nilsson A.; Friebel D. Ambient-Pressure XPS Study of a Ni–Fe Electrocatalyst for the Oxygen Evolution Reaction. J. Phys. Chem. C 2016, 120 (4), 2247–2253. 10.1021/acs.jpcc.5b10931.

[ref35] Favaro M.; Jeong B.; Ross P. N.; Yano J.; Hussain Z.; Liu Z.; Crumlin E. J. Unravelling the Electrochemical Double Layer by Direct Probing of the Solid/Liquid Interface. Nat. Commun. 2016, 7 (1), 12695 10.1038/ncomms12695. 27576762 PMC5013669

[ref36] Favaro M.; Valero-Vidal C.; Eichhorn J.; Toma F. M.; Ross P. N.; Yano J.; Liu Z.; Crumlin E. J. Elucidating the Alkaline Oxygen Evolution Reaction Mechanism on Platinum. J. Mater. Chem. A 2017, 5 (23), 11634–11643. 10.1039/C7TA00409E.

[ref37] Stoerzinger K. A.; Favaro M.; Ross P. N.; Hussain Z.; Liu Z.; Yano J.; Crumlin E. J. Stabilizing the Meniscus for Operando Characterization of Platinum During the Electrolyte-Consuming Alkaline Oxygen Evolution Reaction. Top. Catal. 2018, 61 (20), 2152–2160. 10.1007/s11244-018-1063-6.

[ref38] Stoerzinger K. A.; Favaro M.; Ross P. N.; Yano J.; Liu Z.; Hussain Z.; Crumlin E. J. Probing the Surface of Platinum during the Hydrogen Evolution Reaction in Alkaline Electrolyte. J. Phys. Chem. B 2018, 122 (2), 864–870. 10.1021/acs.jpcb.7b06953. 29166014

[ref39] Carbonio E. A.; Velasco-Velez J.-J.; Schlögl R.; Knop-Gericke A. Perspective—Outlook on Operando Photoelectron and Absorption Spectroscopy to Probe Catalysts at the Solid-Liquid Electrochemical Interface. J. Electrochem. Soc. 2020, 167 (5), 054509 10.1149/1945-7111/ab68d2.

[ref40] Hunter J. D. Matplotlib: A 2D Graphics Environment. Comput. Sci. Eng. 2007, 9 (3), 90–95. 10.1109/MCSE.2007.55.

