# Supplementary material for: Oxidation of Aqueous Phosphorous Acid Electrolyte in Contact with Pt Studied by X-ray Photoemission Spectroscopy
Source: ACS Appl Mater Interfaces. 2023 Oct 27;15(44):51989–99. doi: 10.1021/acsami.3c12557 (PMC10636727; doi:10.1021/acsami.3c12557)
Supplement: Supplementary file 1 — am3c12557_si_001.pdf [file am3c12557_si_001.pdf]

Supporting Information

Oxidation of Aqueous Phosphorous Acid Electrolyte in contact with Pt Studied by X-ray Photoemission Spectroscopy

Romualdus Enggar Wibowo*[,1], Raul Garcia-Diez[1], Tomas Bystron[2], Martin Prokop[2], Marianne van der Merwe[1], Mauricio D. Arce[1,3], Catalina E. Jiménez[1], Tzung-En Hsieh[1], Johannes Frisch[1,4], Alexander Steigert[5], Marco Favaro[6], David E. Starr[6], Regan G. Wilks[1,4], Karel Bouzek[2], Marcus Bär*[,1,4,7,8]

[1]*Dept. Interface Design, Helmholtz-Zentrum Berlin für Materialien und Energie GmbH (HZB), Albert-Einstein-Str. 15, 12489 Berlin, Germany*

[2]*Department of Inorganic Technology, University of Chemistry and Technology Prague. Technicka 5, Prague 6, 166 28, Czech Republic*

[3]*Departamento Caracterización de Materiales, INN-CNEA-CONICET, Centro Atómico Bariloche, Av. Bustillo 9500, S. C. de Bariloche, Rio Negro, 8400, Argentina*

[4]*Energy Materials In-situ Laboratory Berlin (EMIL), HZB, Albert-Einstein-Str. 15, 12489 Berlin, Germany*

[5]*Institute for Nanospectroscopy, Helmholtz-Zentrum Berlin für Materialien und Energie GmbH (HZB), Albert-Einstein-Str. 15, 12489. Berlin, Germany.*

[6]*Institute for Solar Fuels, Helmholtz-Zentrum Berlin für Materialien und Energie GmbH (HZB), Hahn-Meitner-Platz 1, 14109 Berlin, Germany.*

[7]*Department of Chemistry and Pharmacy, Friedrich-Alexander-Universität Erlangen-Nürnberg (FAU). Egerlandstr. 3, 91058 Erlangen, Germany*

[8]*Department of X-ray Spectroscopy at Interfaces of Thin Films, Helmholtz Institute Erlangen-Nürnberg for Renewable Energy (HI ERN), Albert-Einstein-Str. 15, 12489 Berlin, Germany*

Email: enggar.wibowo@helmholtz-berlin.de, marcus.baer@helmholtz-berlin.de

Table of Contents:

1. Topography and surface roughness determination of planar Au and planar Pt electrodes S3

2. Pt black electrode morphology and electrodeposition profile S5

3. P 2p core level measurements for planar Au, planar Pt, and Pt black electrodes in H_3PO_3 and H_3PO_4 .... S6

4. Fitting parameters for the AP-HAXPES on solid crystalline H_3PO_3 and H_3PO_4 acids S7

5. Fitting parameters for the XPS on the acid-treated electrodes S8

6. Open circuit potential (OCP) monitoring of 5 mol dm^{-3} H_3PO_3 in contact with planar Pt, planar Au, and Pt black electrodes S9

7. Cyclic voltammograms of 5 mol dm^{-3} H_3PO_3 and 5 mol dm^{-3} H_3PO_4 solutions with different upper limit potential S12

8. CV of 10 mmol dm^{-3} H_3PO_3 + 0.5 mol dm^{-3} H_3PO_4 using a Pt electrode.................................. S13

9. Validation of the observations at the electrode|electrolyte interface S16

10. Estimation of electrolyte layer thickness on the electrode surface for the *in-situ* AP-HAXPES coupled with the “Dip-and-pull” method S19

11. Fitting parameters and quantification for the P 2p core level in the *in situ* AP-HAXPES coupled with “Dip-and-pull” method.. S20

12. Validation of continuous thin film electrolyte from the probed Pt|thin electrolyte interface to the bulk electrolyte S21

13. Estimation of H_3PO_4 molar fraction resulting from the oxidation of H_3PO_3 by a monolayer of PtO_x S22

14. *In situ* “Dip-and-pull” AP-HAXPES measurements of the Pt-electrode|aqueous H_3PO_3 electrolyte interface S24

15. Comparison of the electrochemical characterization conducted in thin-film only configuration versus fully immersed electrode configuration. S27

16. References S29

1. Topography and surface roughness determination of planar Au and planar Pt electrodes

To verify that the planar Au and planar Pt electrodes used in this study possess a low and comparable surface roughness with each other, atomic force microscopy (AFM) was performed for a detailed insight into these electrode's topography and surface roughness. In addition, electrochemically active surface area (ECSA) determination was made through copper (Cu_{UPD}) and hydrogen (H_{UPD}) underpotential depositions, as it provides information about the whole electrode compared. The AFM and the ECSA determinations of planar Au (left) and planar Pt (right) are shown in **Figure S1**.

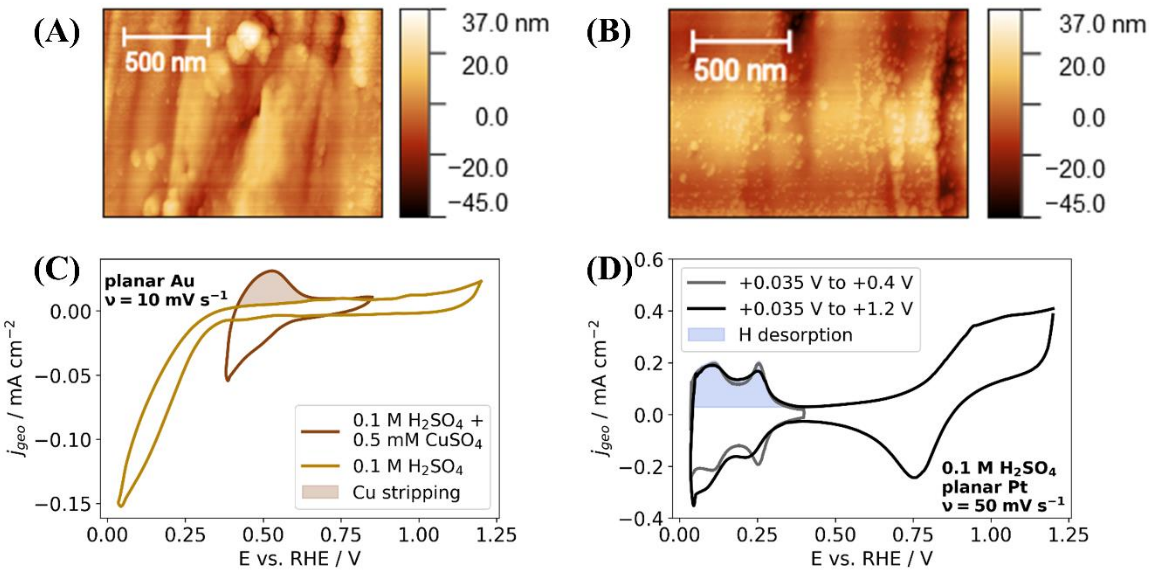

Figure S1. AFM images of **(A)** planar Au and **(B)** planar Pt electrodes. **(C)** Cu_{UPD} on planar Au electrode and **(D)** H_{UPD} of planar Pt electrode for ECSA estimation.

From the AFM scans, comparable surface roughness was observed for both electrodes: planar Au has an average surface roughness of 3.5 nm ± 1.2 nm, while planar Pt has an average surface roughness of 3.5 nm ± 0.6 nm. In the cyclic voltammetry curves of the Cu_{UPD} measurements on the planar Au and Pt thin film electrodes, the shaded regions correspond to the stripping/desorption of the adsorbed underpotentially deposited ions (i.e. Cu^{2+} and H^+ ions for Cu_{UPD} and H_{UPD}, respectively). The ECSA was estimated through the total charge ($Q_{UPD/total}$) determined by the current integration as depicted schematically in the shaded region divided by the scan rate, and subsequently normalized to the specific charge of the underpotentially deposited monolayer of ions (θ^{ref}) on Pt, and summarized in **Table S1**. All measurements for the ECSA determination were conducted with a Pt mesh counter electrode (99.9%, Alfa Aesar) and a reversible hydrogen reference electrode (Mini HydroFlex, Gaskatel). Prior to the measurements, the electrolytes were deaerated by purging with N_2 for 30 minutes.

Table S1. ECSA of planar Au and planar Pt electrodes used in the experiment. A_{geo} is the geometrical area of the electrode in the measurement, $Q_{UPD/total}$ represents the total charge of underdeposited ions, θ^{ref} is specific charge of the underpotentially deposited monolayer of ions on Pt. The roughness factor of the electrode was determined by normalizing the ECSA to the A_{geo}.

Electrode	A_{geo} (cm^2)	$Q_{UPD/total}$ (μC)	θ (μC/cm^2) *ref.*	ECSA (cm^2)	Roughness factor
Planar Au	0.18	74.005	420 [1,2]	0.185	1.023
Planar Pt	0.49	197.51	210 [2,3]	0.940	1.92

Interestingly, the roughness factor determined from electrochemical measurement shows that the planar Pt electrode has a higher roughness factor compared to the Au electrode, unlike the roughness factor determined from the AFM.

2. Pt black electrode morphology and electrodeposition profile

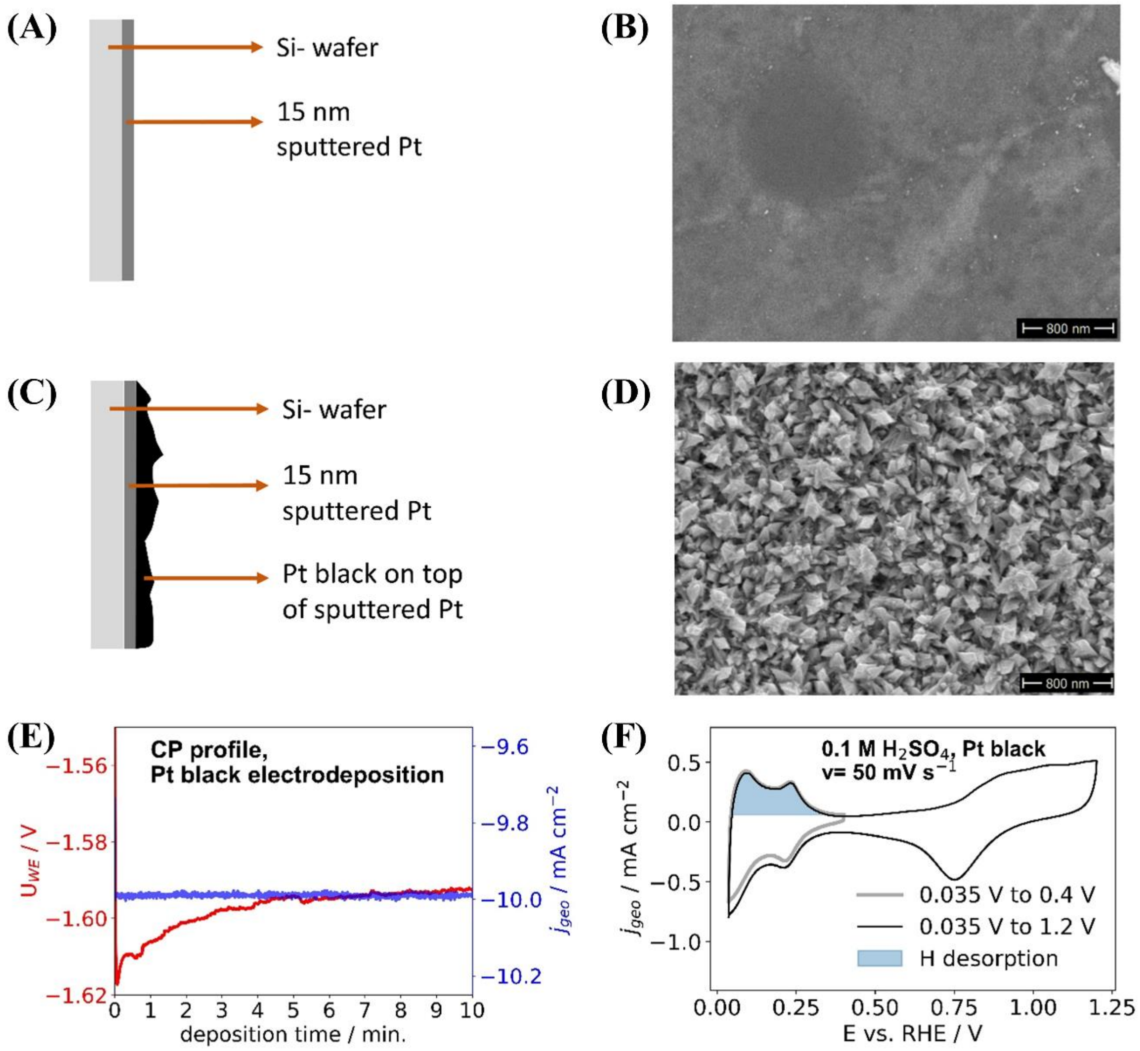

Figure S2. (A) Illustration and **(B)** scanning electron microscopy (SEM) image of the 15 nm thick Pt layer sputtered on top of Si-wafer, as a substrate for the deposition of Pt black working electrode. **(C)** Schematic and **(D)** SEM image of the Pt black deposited on top of the sputtered Pt shown in Fig. S2.A. **(E).** Potential profile and current densities drawn to the working electrodes (i.e. planar 15 nm sputtered Pt on Si-wafer, as shown in **Fig. S2.A**) during Chronopotentiometry (CP), for the electrodeposition of Pt black (**Fig. S2.C**). The experimental details for the preparation of Pt black preparation are given in the experimental section of the main text**. (F)** H_{UPD} of Pt black electrode for the determination of ECSA and roughness factor of the Pt black electrode. CV was recorded with N_2 saturated 0.1 M H_2SO_4, a Pt mesh counter electrode (99.9%, Alfa Aesar), and a reversible hydrogen reference electrode (Mini HydroFlex, Gaskatel) , with a scan rate of 50 mV s^{-1}.

From the hydrogen desorption of the UPD, ECSA was determined ~7.52 cm^2. The roughness factor of the Pt black electrode was estimated from the ratio of ECSA to the geometrical area of the electrode used for the measurement (A_{geo} ~1.26 cm^2), in which the electrode displayed a roughness factor of ~5.97.

3. P 2p core level measurements for planar Au, planar Pt, and Pt black electrodes in H_3PO_3 and H_3PO_4

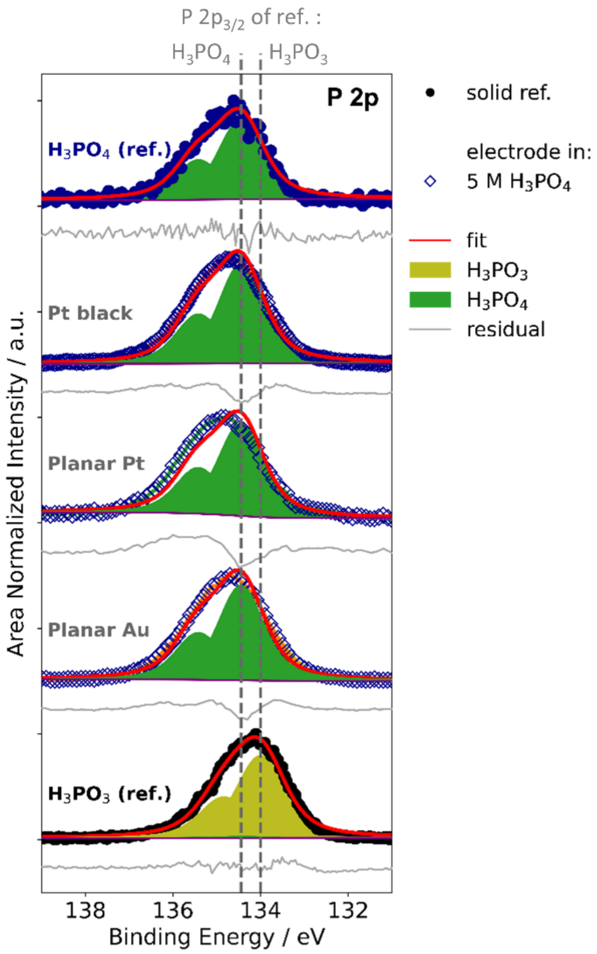

Figure S3. X-ray photoelectron spectroscopy (XPS) on P 2p core level of Au and Pt electrode previously treated in 5 mol dm^{-3} H_3PO_4, as given in **Figure 2** in the main text, along with the curve fitting. The dashed lines correspond to the binding energies of P $2p_{3/2}$ of the solid H_3PO_3 and solid H_3PO_4 references. The peak contribution almost exclusively emerges from H_3PO_4, similar to the solid H_3PO_4 crystalline reference, indicating that the solution is stable on the electrodes. XPS on the acid-treated electrode was performed with Mg K_α excitation (1253.56 eV) at a pressure < 5×10^{-8} mbar.

4. Fitting parameters for the AP-HAXPES on solid crystalline H_3PO_3 and H_3PO_4 acids

Table S2. Fitting parameters for the ambient-pressure hard photoelectron spectroscopy (AP-HAXPES) of the P 2p core level of the solid crystalline H_3PO_3 and H_3PO_4 references, as given in **Figure 2**, in the main text. The P 2p doublet separation was kept at 0.84 eV for solid H_3PO_3 and 0.97 eV for solid H_3PO_4, following optimization between 0.7 eV – 1.0 eV.

**Compounds**	**P $2p_{3/2}$**		**P $2p_{1/2}$**	
	**B.E. of peak maxima (eV)**	**FWHM (eV)**	**B.E. of peak maxima (eV)**	**FWHM (eV)**
**solid cryst. H_3PO_3 (ref.)***	133.99	0.4	134.88	0.4
**solid cyrst. H_3PO_4 (ref.)***	134.47	0.4	135.45	0.4

*The Voigt model parameters (σ,γ) used for the fitting process are (0.37, 0.37) and (0.35, 0.35), for solid H_3PO_3 and solid H_3PO_4 references, respectively.

5. Fitting parameters for the XPS on the acid-treated electrodes

Table S3. Peak area and the calculated molar ratio of species from the XPS of the P 2p core level of the different substrates (planar Au and planar Pt) dipped into H_3PO_3 or H_3PO_4 solutions, as given in **Figure 2** in the main text. The binding energy of the peak, FWHM, and Voigt model parameters (σ,γ) of solid reference are given in **Table S2)**.

**Compounds/ Acid-treated electrodes**	**H_3PO_3**		**H_3PO_4**		**Molar ratio of each species to the total amount**	
	**P $2p_{3/2}$ peak area (a.u)**	**P $2p_{1/2}$ peak area (a.u.)**	**P $2p_{3/2}$ peak area (a.u)**	**P $2p_{1/2}$ peak area (a.u.)**	**H_3PO_3 : (H_3PO_3 + H_3PO_4)**	**H_3PO_4 : (H_3PO_3 + H_3PO_4)**
**solid cryst. H_3PO_3 (ref.)**	1.29	0.65	0.06	0.03	0.96	0.04
**planar Au in 5 mol dm^{-3} H_3PO_3**	0.94	0.47	0.55	0.27	0.63	0.37
**planar Pt in 5 mol dm^{-3} H_3PO_3**	0.65	0.32	0.83	0.42	0.44	0.56
**Pt black in 5 mol dm^{-3} H_3PO_3**	0.24	0.12	1.26	0.63	0.16	0.84
**solid cryst. H_3PO_4 (ref.)**	0.00	0.00	1.21	0.61	0.00	1.00
**planar Au in 5 mol dm^{-3} H_3PO_4**	0.12	0.06	1.37	0.69	0.08	0.92
**planar Pt in 5 mol dm^{-3} H_3PO_s**	0.00	0.00	1.40	0.70	0.00	1.00
**Pt black in 5 mol dm^{-3} H_3PO_s**	0.03	0.01	1.47	0.74	0.02	0.98

6. Open circuit potential (OCP) monitoring of 5 mol dm^{-3} H_3PO_3 in contact with planar Pt, planar Au, and Pt black electrodes

To gain further insights into the oxidation of aqueous H_3PO_3 on the planar Pt, planar Au, and Pt black electrodes, the open circuit potential (OCP) of these electrodes in 5 mol dm^{-3} H_3PO_3 solution was continuously monitored for ~15 hours. Prior to the experiments, the electrode was cleaned by immersion in 2 mol dm^{-3} H_2SO_4 for 6 hours, followed by thorough rinsing using Milli-Q water. Subsequently, the electrodes were activated in N_2 purged 5 mol dm^{-3} H_3PO_3 electrolyte through potential cycling within the water stability window: +0.05 V and +1.0 V vs. RHE, with a scan rate of 50 mV s^{-1}, until no changes were observed in the region corresponding to the hydrogen desorption region (around +0.05 V to +0.4 V during positive-going potential sweep). Following the activation, the OCP was monitored in a 5-second intervals for approximately 15 hours until a steady state response was achieved. Throughout the OCP recording, the electrolyte was constantly stirred (rotation speed of 380 rpm, using IKA C-MAG HS7, magnetic stirrer) to ensure sufficient intermixing of the H_3PO_3 electrolyte in the proximity of the Pt electrode, where the surface-catalyzed oxidation might occur. The stirring reduces the time required for H_3PO_3 to reach the electrode from the bulk solution and enables a qualitative analysis within a shorter duration of OCP monitoring. This is because changes in the OCP are indicative of alterations in the electrode|electrolyte interface, which may result from reactions at the interface (such as the oxidation of H_3PO_3 to H_3PO_4 at the Pt surface). The OCP was recorded using a BioLogic SP-300 double channel potentiostat, with a Pt mesh (99.9%, Alfa Aesar) counter electrode, a reversible hydrogen reference electrode (Mini HydroFlex, Gaskatel), and 40 ml of electrolyte volume. The working electrodes possess a geometrical area of ~0.875 cm^2. The recorded OCP data of 5 mol dm^{-3} H_3PO_3 on the planar Pt, planar Au, and Pt black electrodes are presented in **Figure S4**.

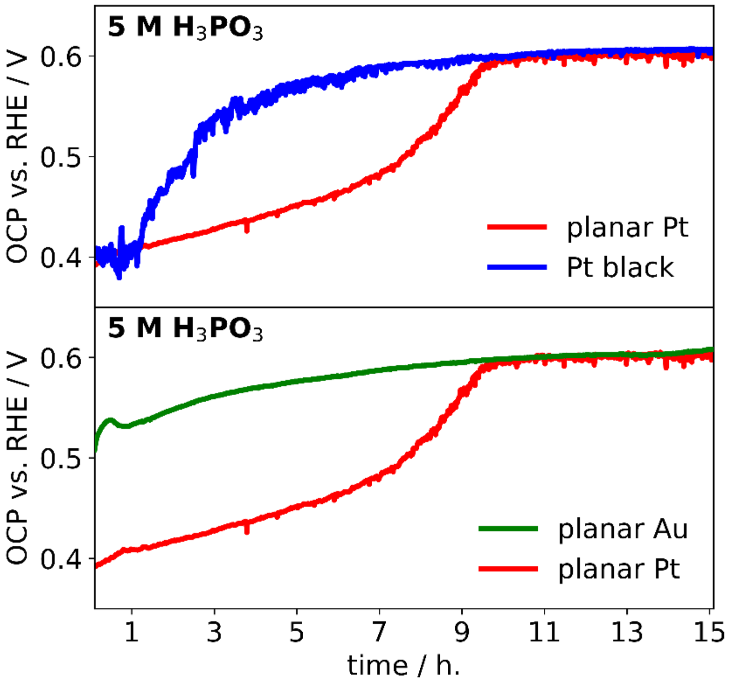

Figure S4. OCP monitoring of 5 mol dm^{-3} H_3PO_3 on Pt electrodes of different surface roughness (**Top Panel**): planar Pt and rougher Pt black; and on different planar metal electrodes **(Bottom panel)**: planar Au and planar Pt. Experiments were performed with a constant stirring of 380 rpm. The displayed OCP values represent averages over a 50 seconds-interval (10 data points).

Based on **Figure S4,** it is evident that the OCP of each electrode increases during recording, likely indicating the chemical oxidation of H_3PO_3 to H_3PO_4. This observation aligns with blocking the electrode surface by H_3PO_4 over time, as seen in **Figure 1.D** of the main text for Pt/C catalysts in the H_3PO_3 electrolyte. Notably, the rate of OCP change is faster for the (rough) Pt black compared to the planar Pt electrode. Among the planar electrodes, the OCP change for the planar Au electrode is comparatively smaller than that for the planar Pt electrode. The distinct rate of OCP change correlates well with the XPS-derived H_3PO_4 molar ratio of H_3PO_4 from the 5 mol dm^{-3} H_3PO_3 acid-treated electrodes shown in **Figure 2** of the main text (i.e., Pt black electrode shows higher yields of H_3PO_4 molar ratio compared to planar Pt and planar Au electrodes). This further indicates the catalytic role of Pt in facilitating the oxidation of H_3PO_3 to H_3PO_4 through H_2O, as discussed in the main text and represented by Eq.1.

Please note that since the OCP corresponds to a mixed potential at which the sum of all anodic currents is equal to the sum of absolute values of all cathodic currents, the OCP reflects the rate of the individual surface processes taking place at the electrode surface. These rates are influenced by numerous interconnected conditions and phenomena, and at the initial stage of the OCP, the conditions are quite complex. Hence, in the present discussion, focus is given to the general trend of OCP which suggests blocking of the electrodes due to the oxidation of H_3PO_3 on the electrode surface, as previously discussed.

Additionally, it is important to note that although the change in OCP provides quantitative information on the chemical oxidation of aqueous H_3PO_3 to H_3PO_4 to a certain extent, it does not allow a precise estimation of the reaction rate. The limitation arises because OCP provides the information from the state of the electrode|electrolyte interface, which is influenced by various factors such as the rate and type of the electrode reactions, as well as the mass transport of H_3PO_3 from the bulk solution to the interface. As the impact of these factors remains unknown, in this study OCP is solely used to gain qualitative insights into the chemical oxidation process and its subsequent effects on Pt electrodes.

7. Cyclic voltammograms of 5 mol dm^{-3} H_3PO_3 and 5 mol dm^{-3} H_3PO_4 solutions with different upper limit potential

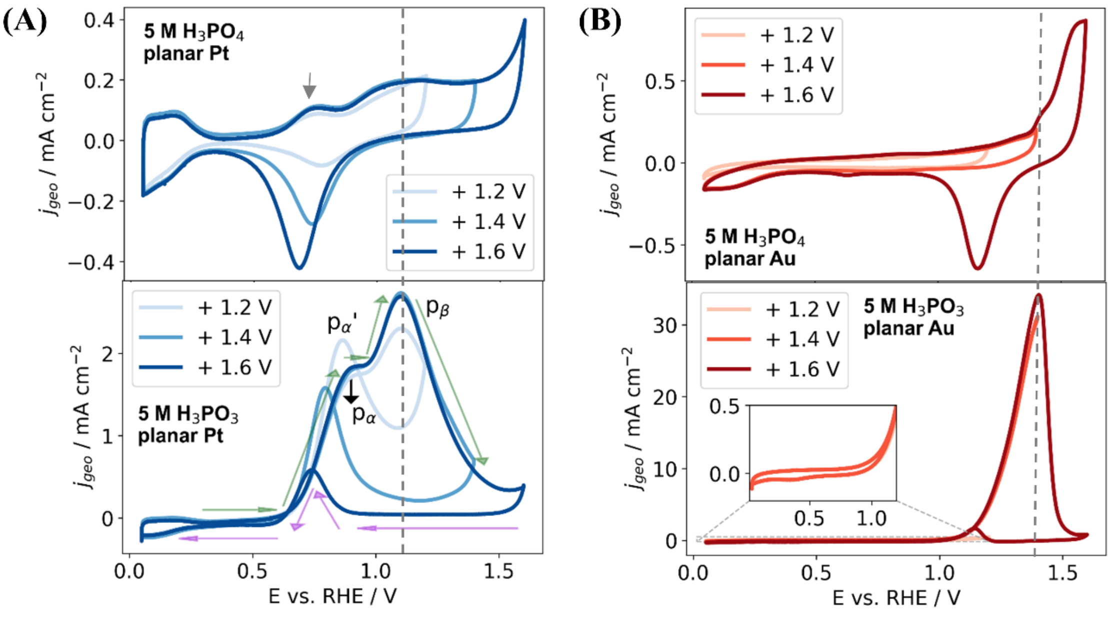

Figure S5. Cyclic voltammograms (CV) of **(A)** a planar Pt and **(B)** planar Au working electrodes in **(top):** 5 mol dm^{-3} (5 M) H_3PO_4 and (**bottom):** in 5 mol dm^{-3} (5 M) H_3PO_3. All CVs were recorded with the starting potential of +0.05 V vs. RHE, using the scan rate of 50 mV s^{-1}. The current response during the positive-going potential sweep in panel **(A, bottom)** is shown with a green arrow, while the violet arrow illustrates the current response during the negative-going potential sweep. The inset graph in panel **(B, bottom)** shows a magnified look at the lower potential region with the lower current response. Each CV was recorded with three different upper reversal potentials (+1.2 V, +1.4 V, and +1.6 V vs. RHE). Between each CV, several scans were taken until steady-state voltammograms were obtained and then recorded. The small gray arrow on **(A, top)** indicates H_3PO_3 oxidation peak (i.e. indicating minor contamination of the solution by H_3PO_3). Gray dashed lines indicate the peak potential of H_3PO_3 oxidation on the surface oxides for each planar electrode, as shown in the bottom figure of **Figure S4.A** and **Figure. S4.B**.

8. CV of 10 mmol dm^{-3} H_3PO_3 + 0.5 mol dm^{-3} H_3PO_4 using a Pt electrode

For further evidence that the observed peak pα (see **Figure 3** of the main text) indeed corresponds to the electrochemical oxidation of H_3PO_3 to H_3PO_4 in the presence of Pt. The CVs of the Pt electrode were recorded in an aqueous H_3PO_4-based electrolyte containing a relatively low concentration of H_3PO_3, within the potential window of +0.05 V to +1.2 V vs. RHE. The experiments are performed as follows: H_3PO_4 electrolyte with the concentration of 0.5 mol dm^{-3} H_3PO_4 was prepared by diluting crystalline H_3PO_4 (99.99 wt.%, Merck) with Milli-Q water until the concentration of 0.5 mol dm^{-3} H_3PO_4 is achieved. Subsequently, the electrolyte was deaerated by purging with N_2 gas. Then, the Pt black working electrode was activated by conducting a CV on deaerated 0.5 mol dm^{-3} H_3PO_4 in the potential range of +0.05 V_{RHE} to +1.0 V_{RHE} (i.e., in the water stability window) with a potential scan rate of 50 mV s^{-1}, until a steady state voltammogram was obtained. A Pt mesh counter electrode (99.9%, Alfa Aesar), a reversible hydrogen reference electrode (Mini HydroFlex, Gaskatel), and a Bilogic SP300 double channel potentiostat were used for this experiment. Following the activation, CV measurements in 0.5 mol dm^{-3} H_3PO_4 in the potential range of +0.05 V_{RHE} to +1.2 V_{RHE} were recorded, as reference of Pt electrode CV response in pure aqueous H_3PO_4 electrolyte. Subsequently, H_3PO_3 (99%, Merck) was added into the 0.5 mol dm^{-3} H_3PO_4 to achieve a 10 mmol dm^{-3} solution and it was stirred at 380 rpm for 15 minutes (using IKA C-MAG HS7, magnetic stirrer) before starting continuous CV measurements in the potential range of +0.05 V to +1.2 V with a scan rate of 50 mV s^{-1} for an extended period of 11,000 cycles (i.e., ~6 days). The CVs are shown in **Figure S6.A**. By performing the CV under these conditions, it is possible to observe whether peak pα indeed corresponds to the oxidation of H_3PO_3 to H_3PO_4. If peak pα indeed corresponds to the oxidation of H_3PO_3, it is expected that over the cycling process, **(i)** the magnitude of peak pα should decrease, and (**ii)** the transition of a voltammogram response from a CV characteristic for H_3PO_3 to that typical for H_3PO_4 might be observed.

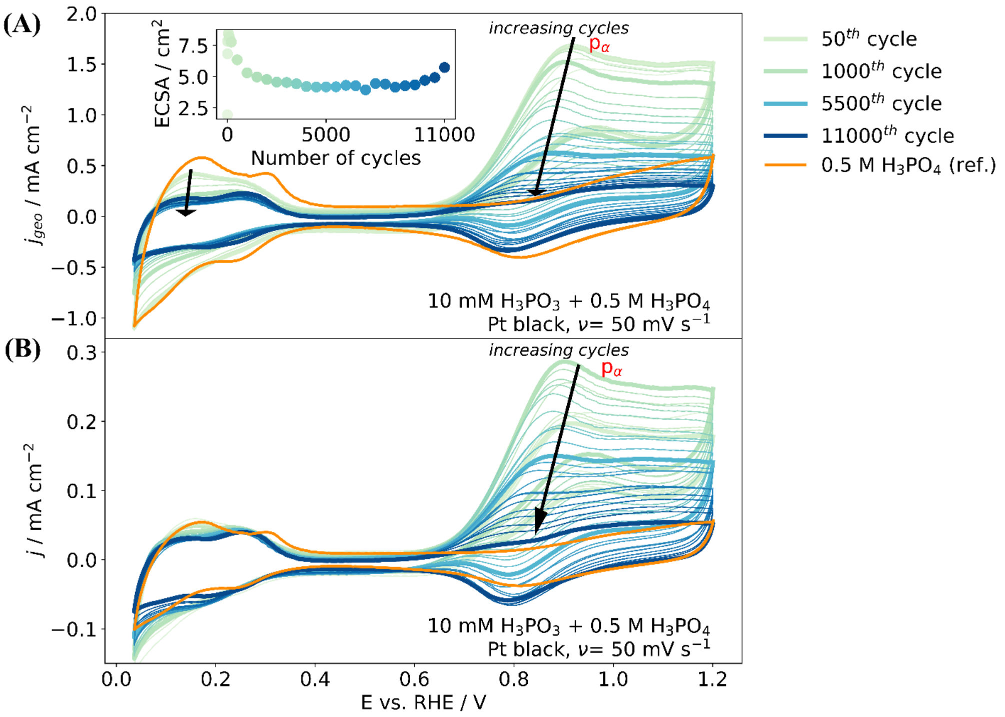

Figure S6. CV of 10 mmol dm^{-3} H_3PO_3 + 0.5 mol dm^{-3} H_3PO_4 on Pt black electrode, recorded for 11,000 cycles with a scan rate of 50 mV s^{-1}. Individual CVs are shown for every 500 cycles, along with the CV of 0.5 mol dm^{-3} H_3PO_4 on the same electrode, for comparison. In **(A)** the recorded current is normalized to the geometrical area of the working electrode, while in **(B)** the recorded current is normalized to the ECSA of the working electrode.

As depicted in **Figure S6.A,** with an increasing number of cycles, two notable changes emerge: **(i)** A decrease of current response in the H-adsorption region (i.e. +0.05 V to +0.4 V in the positive-going potential sweep), indicating a decrease of ECSA of the Pt electrode, likely due to smoothening of Pt black surface over prolonged potential cycling and **(ii)** a decrease of the pα peak current density corresponding to the electrochemical oxidation of H_3PO_3 with an increasing number of cycles. This trend continues until the voltammogram progressively attains the well-known CV features for a Pt electrode in aqueous 0.5 mol dm^{-3} H_3PO_4 electrolyte. It is important to note that the decrease of the pα peak current is also observed when normalizing the recorded CV current with the ECSA of Pt (see **Figure 6.B**). The ECSA was estimated by integrating the CV current response in the potential window between +0.05 V to +0.4 V during the positive potential sweep, and subsequently normalizing it by the scan rate and the specific charge of the underpotentially deposited monolayer of ions (θ^{ref}) on Pt (210 µC cm^{-1}, see Ref. [2,3]). The concurrent decrease of the pα peak and the transition of CV response from the characteristic CV of Pt in aqueous H_3PO_3 (e.g., from 50th cycles, similar to **Figure 5.A** for H_3PO_3 electrolyte) to the characteristic CV of Pt in aqueous H_3PO_4 (e.g. from 11,000th cycles, similar to **Figure 5.A** for H_3PO_4 electrolyte), provide compelling evidence that peak pα indeed corresponds to the electrochemical oxidation of H_3PO_3 to H_3PO_4. This result also shows that under the experimental

condition, the oxidation of H_3PO_3 to H_3PO_4 is irreversible. Additionally, the slight increase in observed ECSA after a high number of cycles (≥ 10,000 cycles) might suggest that following the significant conversion of H_3PO_3 to H_3PO_4 in the electrolyte, previously blocked Pt surfaces are partially re-activated.

9. Validation of the observations at the electrode|electrolyte interface

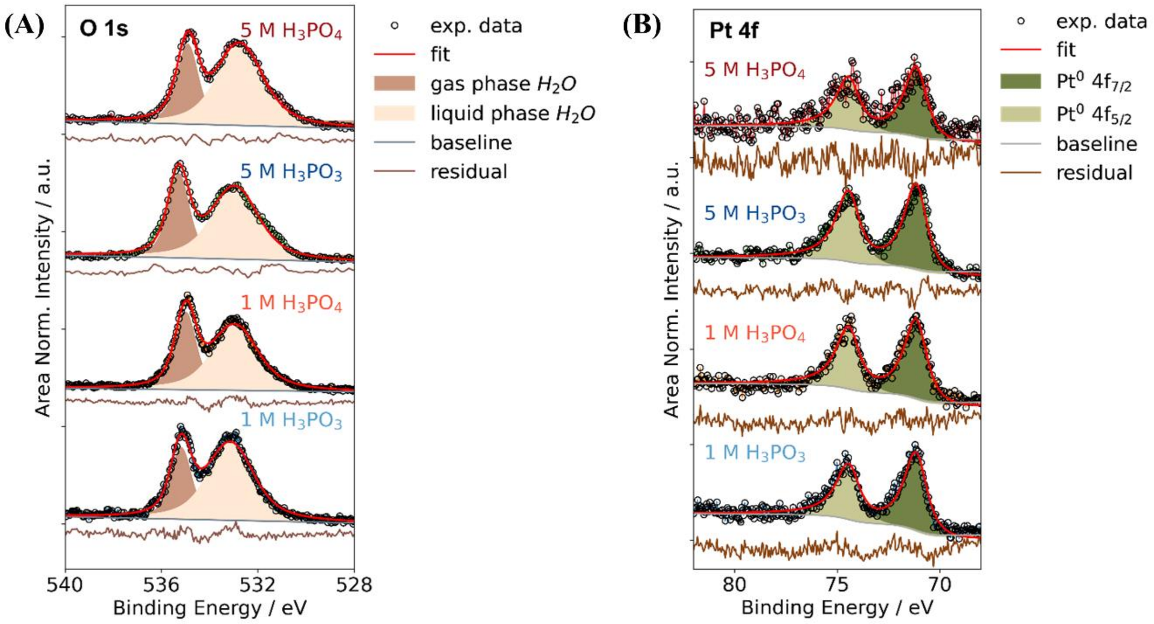

Figure S7. *In situ* AP-HAXPES coupled with the "Dip-and-pull" method, conducted on the Pt black|electrolyte interface on the: (**A)** O 1s core level and **(B)** Pt 4f core level. Measurements were performed at the same positions in which P 2p core level was measured (**Figure 4** in the main text). All measurements were recorded with the excitation energy of 3 keV. Measurements with 1 mol dm^{-3} electrolytes were made at the pressure of 22 mbar, while measurements with 5 mol dm^{-3} solution were performed at the pressure of 18 mbar.

In the measurement positions, the signal arising from the electrode (i.e. Pt 4f core level from Pt black electrode) and electrolyte (i.e. liquid phase water in the O 1s core level, from the aqueous electrolyte) could be observed. This shows that the region probed during the AP-HAXPES measurements, indeed corresponds to the electrode|thin layer electrolyte interface. All measurements were conducted at the open circuit potential. Fitting parameters are given in **Table S4** and **Table S5** in the following.

Table S4. Fitting parameters for the AP-HAXPES of the O 1s core level of the Pt black|electrolyte interface shown in **Figure S5**. Peaks were fitted with a Voigt profile and a Shirley background. FWHM* and Voigt model parameters (σ, γ) of each species (gas phase H_2O and liquid phase H_2O) were kept constant during the fitting procedure.

**Electrolyte**	**Gas phase H_2O**		**Liquid phase H_2O**	
	**B.E. of peak maxima (eV)**	**Peak area (a.u.)**	**B.E. of peak maxima (eV)**	**Peak area (a.u.)**
**1 mol dm^{-3} H_3PO_3***	535.19	1.84	533.13	4.77
**1 mol dm^{-3} H_3PO_4***	534.99	2.37	532.94	4.65
**5 mol dm^{-3} H_3PO_3***	535.29	3.10	532.96	6.04
**5 mol dm^{-3} H_3PO_4***	534.91	2.06	532.74	5.11

*Full-width half maxima (FWHM) for the fitting process were kept constant at: 0.97 eV and 2.31 eV, for gas phase H_2O and liquid phase H_2O, respectively. Voigt model parameters (σ, γ) for the fitting are: (0.26, 0.26) and (0.64, 0.64) for the gas phase H_2O and liquid phase H_2O, respectively

Table S5. Fitting parameters for the AP-HAXPES on the Pt 4f core level in the Pt black|electrolyte interface given in **Figure S5**. The asymmetric Pt 4f peaks were fitted using a Doniach–Šunjić profile and Shirley background, for a meaningful fit with metallic core level. The area of Pt $4f_{5/2}$ component was kept to 75 % of the area of Pt $4f_{7/2}$ component [4]. Moreover, with the assumption that the same species should arise under all the different conditions (i.e. Pt^0), the position of the peak, FWHM, and the Doniach–Šunjić model parameters (σ and γ) were kept constant for the fitting.

**Electrolyte**	**Pt^0 (Pt $4f_{7/2}$)**		**Pt^0 (Pt $4f_{5/2}$)**	
	**B.E. of peak maxima (eV)**	**Peak area (a.u.)**	**B.E. of peak maxima (eV)**	**Peak area (a.u.)**
**1 mol dm^{-3} H_3PO_3***	71.09	3.50	74.39	2.20
**1 mol dm^{-3} H_3PO_4***		3.71		2.57
**5 mol dm^{-3} H_3PO_3***		3.95		2.91
**5 mol dm^{-3} H_3PO_4***		2.21		1.47

*The FWHM was kept constant at 1.40 eV for both Pt $4f_{7/2}$ and Pt $4f_{5/2}$ components. The Doniach–Šunjić model parameters (σ, γ) were (0.75, 0.11)

10. Estimation of electrolyte layer thickness on the electrode surface for the *in-situ* AP-HAXPES coupled with the "Dip-and-pull" method

The estimation of the electrolyte layer thickness was made by deriving the Lambert-Beer equation, as presented in Ref. [5], which takes into account: (i) the intensity of photoelectron flux generated from the electrode, which has been attenuated by the electrolyte layer, (ii) the intensity of photoelectron flux arising from the attenuating electrolyte, as well (iii) the inelastic mean free path (IMFP) of the photoelectrons, and (iv) the density of the electrode and electrolyte. This gives the following equation:

$$\boldsymbol{t_{H_2O} = \lambda_{H_2O}\left(1 + \frac{I_{H_2O}}{I_{Pt}}\frac{N_{Pt}\ \lambda_{Pt}}{N_{H_2O}\ \lambda_{H_2O}}\right)} \qquad \text{Eq. S1.}$$

t_{H_2O} is the thickness of the electrolyte layer (in nm). λ_x is the inelastic mean free path (IMFP) of the photoelectron for substance x (in nm). The value for λ_{H_2O} was adapted from the calculation by Emfietzoglou and Nikjoo [6] (~8.34 nm), while the value for λ_{Pt} was determined by the TPP2M equation [7] (29.48 nm). I_x corresponds to the peak area of component *x*. The I_{H_2O} was integrated from peak area of liquid phase H_2O in the recorded O 1s spectrum, while I_{Pt} was integrated from peak area of Pt $4f_{7/2}$ core level under the same conditions. N_x is the number of atoms/molecules per unit volume for the substance *x*. For N_{H_2O} and N_{Pt}, 33.4 nm^{-3} and were 66.19 nm^{-3} used, respectively, as used by Ref. [8]. The estimated thicknesses of the thin electrolyte layers found at the probed Pt|aqueous electrolyte interfaces across different electrolyte solutions have been detailed in **Table S6**.

Table S6. Electrolyte layer thickness ($t_{electrolyte} = t_{H2O}$) on the electrode surface in the *in situ* AP-HAXPES combined with the "Dip-and-pull" method

Electrolyte	$t_{electrolyte}$ (nm)
**1 mol dm^{-3} H_3PO_3**	22
**1 mol dm^{-3} H_3PO_4**	24
**5 mol dm^{-3} H_3PO_3**	24
**5 mol dm^{-3} H_3PO_4**	28

It is important to note, that the estimation method employed here is typically used for planar electrodes and may not be as precise for electrodes with higher surface roughness, such as Pt black. Consequently, the estimated electrolyte layer thicknesses for rougher electrodes carry larger uncertainties when compared to planar electrodes. Nevertheless, despite these uncertainties, this estimation is conducted to provide an approximate value of the electrolyte layer thickness, as the oxidation of aqueous H_3PO_3 on Pt is expected to be correlated with the thickness of the electrolyte layer. It is worth noting that despite the potential limitations for rougher electrodes, the estimated thickness of the electrolyte layer in this study aligns well with the values reported in previous studies [8–10,10–12]

11. Fitting parameters and quantification for the P 2p core level in the *in situ* AP-HAXPES coupled with "Dip-and-pull" method

Table S7. Fitted P 2p core level areas for the AP-HAXPES the Pt black|electrolyte interface as given in **Figure 4.A** and **4.B** in the main text. The B.E. of the peak maxima, FWHM, and Voigt model parameters (σ, γ) were kept similar to the solid reference (as given in **Table S2**).

**Electrolyte**	**Peak areas (a.u.)**				**Molar fraction of each species to the total amount ($H_3PO_3 + H_3PO_4$)**	
	**H_3PO_3**		**H_3PO_4**			
	**P $2p_{3/2}$**	**P $2p_{1/2}$**	**P $2p_{3/2}$**	**P $2p_{1/2}$**	**H_3PO_3**	**H_3PO_4**
**1 mol dm^{-3} H_3PO_3**	0.17	0.09	1.17	0.58	0.13	0.87
**1 mol dm^{-3} H_3PO_4**	0.00	0.00	0.96	0.48	0.01	0.99
**5 mol dm^{-3} H_3PO_3**	0.31	0.16	0.75	0.38	0.29	0.70
**5 mol dm^{-3} H_3PO_4**	0.00	0.00	0.99	0.50	0.01	0.99

12. Validation of continuous thin film electrolyte from the probed Pt|thin electrolyte interface to the bulk electrolyte

To verify that the probed spot at the electrode|electrolyte was connected to the bulk electrolyte solution, O 1s and Pt 4f spectra of the electrode|thin layer electrolyte were recorded during potential application of +0.05 V and +1.0 V vs. RHE to the working electrode. Subsequently, the shift of the spectra binding energy (B.E.) during these potential applications was compared to the expected ones.

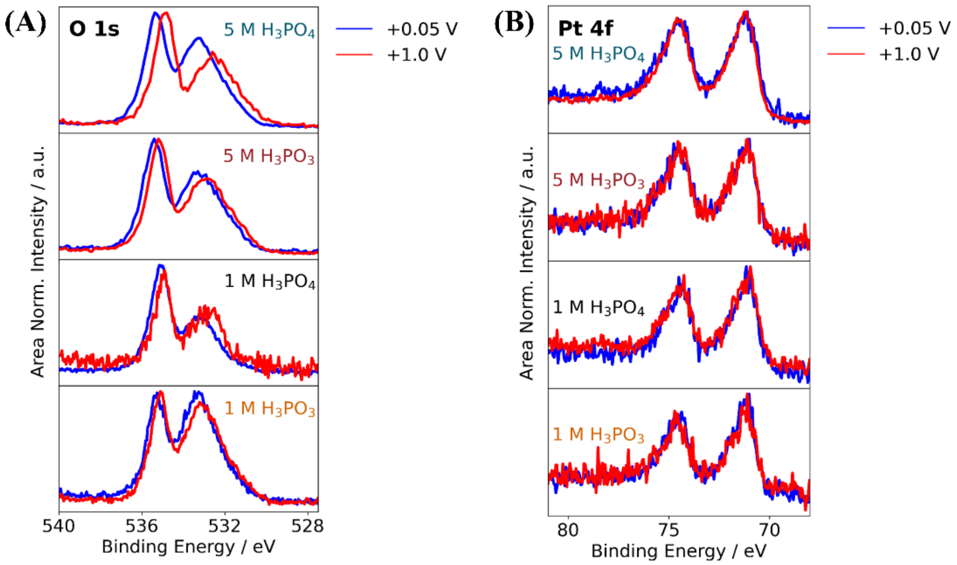

Figure S8. *In situ* AP-HAXPES coupled with the "Dip-and-pull" method was conducted on the Pt black|electrolyte interface during potential application of +0.05 V and +1.0 V vs. RHE, measuring: **(A)** the O 1s core level and **(B)** the Pt 4f core level. All measurements were recorded with an excitation energy of 3 keV. Measurements with 1 mol dm^{-3} electrolytes were made at a pressure of 22 mbar, while measurements with the 5 mol dm^{-3} solution were performed at a pressure of 18 mbar.

As shown in **Figure S8**, there is a spectral shift observed in the O 1s core level, while no shift is observed in the Pt 4f spectra. Due to the potential applied to the Pt electrode, a binding energy shift of the O 1s spectra arising from the electrolyte corresponding to the applied potential was observed. If the probed spot was disconnected from the bulk solution electrolyte, or if the electrolyte layer consist of many droplets, such a shift would be absent [9]. It is important to note, that since the electrode possesses common ground with the electron analyzer, the Pt 4f core level energy does not shift like the O 1s spectra [9]. For the measurement with 5 mol dm^{-3} H_3PO_4 solution, there is a drop in peak intensity corresponding to the liquid phase water (~532.0 eV) between the application of +0.5 V and +1.0 V. This could indicate that the electrolyte layer was slightly unstable and thinned out during the measurement, although a good connection to the bulk electrolyte solution was still maintained. For all the other compounds the intensity of the peak assigned to the liquid phase H_2O remains comparable for the +0.5 V and +1.0 V measurements (i.e. the thin electrolyte layer remains stable).

13. Estimation of H_3PO_4 molar fraction resulting from the oxidation of H_3PO_3 by a monolayer of PtO_x

Since the possibility of an oxidized Pt monolayer (i.e. PtO_x) at the investigated electrode during the *in situ* AP-HAXPES measurement cannot be excluded, an estimation of the H_3PO_4 molar fraction resulting from the oxidation of H_3PO_3 by a such hypothetical PtO_x monolayer was made. By comparing the theoretical molar fraction of H_3PO_4 resulting from the oxidation of H_3PO_3 by a monolayer of PtO_x with the observed H_3PO_4 molar fraction observed in the AP-HAXPES measurement (given in **Table S7**), further insight into the oxidation process occurring during the *in situ* AP-HAXPES could be made.

For this estimation, firstly the number of H_3PO_3 molecules inside the electrolyte volume probed during AP-HAXPES was estimated. The estimation was performed by using Eq. S2.

$$no.\,of\ H_3PO_3\ molecules = (V_{H_3PO_3} \times c)\,N_A = \{\,(A_{beam\ spot} \times\ t_{electrolyte})\ c\,\}\,N_A \qquad \text{Eq. S2.}$$

$V_{H_3PO_3}$ represents the probed H_3PO_3 electrolyte volume, estimated from electrolyte thickness, $t_{electrolyte}$ (given in **Table S6**), and the probed area given by the beam spot, $A_{beam\ spot}$: approximately 400 µm × 700 µm. **Figure S9** illustrates the model used for estimating the number of H_3PO_3 molecules in the probed electrolyte volume. c in the Eq. S2 corresponds to the concentration of the solution (in Molar, i.e. mol dm^{-3}), and N_A is Avogadro's constant (N_A= 6.022 × 10^{23} mol^{-1}).

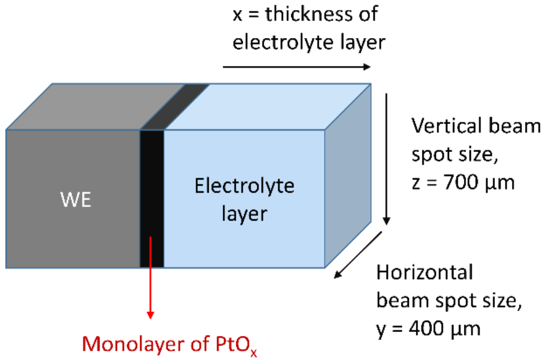

Figure S9. Illustration of the model for estimation of H_3PO_4:(H_3PO_3+H_3PO_4) molar ratio resulting from oxidation from a monolayer of PtO_x.

Subsequently, the number of PtO_x forming a monolayer on the electrode surface probed in the AP-HAXPES was also estimated by Eq. S3.

$$number\ of\ PtO_x\ covering\ monolayer\ surface = \left(\frac{\theta_{PtO_x} N_A}{F}\right) \times \left(A_{beam\ spot}\ rf_{WE}\right) \qquad \text{Eq. S3}$$

θ_{PtOx} corresponds to the specific surface charge of a PtO monolayer (420 µC cm^{-2}, according to ref. [13]), $A_{beam\ spot}$ is the probed surface area given by the beam spot (approximately 400 µm × 700 µm), F represents the Faraday constant (F = 9.648 × 10^4 C mol^{-1}), and rf_{WE} corresponds to the roughness factor of the working electrode (rf_{WE}~ 5.97, see detail in section 2, **Figure S2**). Through Eq. S3, the number of PtO covering the whole surface area of the beam spot was estimated to be ~4.38 × 10^{13}.

Finally, an estimation of the H_3PO_4 molar fraction is made through the number of PtO in probed monolayer surface, to the number of H_3PO_3 molecules in the probed electrolyte volume (assuming each PtO is oxidizing one H_3PO_3 molecule). This estimated value is given in **Table S8**.

Table S8. Estimation of H_3PO_4 molar fraction resulting from the oxidation of H_3PO_3 by a monolayer PtO_x.

**Electrolyte**	**Number H_3PO_3 molecules in the probed volume**	**H_3PO_4 fraction due to the H_3PO_3 oxidation by a monolayer of PtO_x (%)**
**1 mol dm^{-3} H_3PO_3**	3.62×10^{13}	120.31
**5 mol dm^{-3} H_3PO_3**	2.08×10^{14}	21.01

As shown in **Table S8**, while a monolayer of hypothetical PtO_x may fully oxidize 1 mol dm^{-3} H_3PO_3, the H_3PO_4 molar fraction in the 5 mol dm^{-3} H_3PO_3 due to the oxidation of H_3PO_3 by a PtO_x monolayer is smaller compared to the H_3PO_4 molar fraction observed by the AP-HAXPES (see fractions in **Table S3**). This indicates that the oxidation cannot be explained solely by the presence of PtOx, and other processes must be responsible for the oxidation of the H_3PO_3 (e.g. oxidation of H_3PO_3 through H_2O as proposed in the main text). Furthermore, it is important to note that, this assumption is made for an extreme situation, in which the generated H_3PO_4 stays on the electrode surface and it does not diffuses away from the proven volume. However, given the experimental duration in the probed spot (~1.5 hrs), diffusion likely occurs, which means that less H_3PO_4 produced from this phenomenon should be observed. Moreover, in the event that oxidation by PtO_x occurs in a very short timescale, during the "dipping" of the electrode in the electrolyte (i.e. before "pulling" the electrode up to form the electrode|thin electrolyte layer for the *in situ* AP HAXPES), the H_3PO_3 might already by oxidized in this process. Hence, at the time of measurement, the H_3PO_4 formed by this process might already diffuse away. As result, even less influence of oxidation by PtO_x should be observed in this case.

14. *In situ* "Dip-and-pull" AP-HAXPES measurements of the Pt-electrode|aqueous H_3PO_3 electrolyte interface

To verify the reproducibility of the obtained results, two data sets of *in situ* "Dip-and-pull" AP-HAXPES measurements of the Pt black|aqueous H_3PO_3 interface are compared. These measurements were performed under OCP conditions using both, 1 mol dm^{-3} H_3PO_3 and 5 mol dm^{-3} H_3PO_3 electrolytes. The same experimental setup detailed in the experimental section of the main text is used for both experiments. Furthermore, apart from the measurements made at the Pt-electrode|aq. H_3PO_3 interface, additional "Dip-and-pull" *in situ* AP HAXPES were also performed in a slightly thicker part of the electrolyte layer. In this region, the AP-HAXPES signal arising from the electrode (i.e., the Pt 4f core level) was no longer observable. This measurement aimed to provide insights into the influence of electrolyte layer thickness on the state of the H_3PO_3 electrolyte. Results from these experiments are shown in **Figure S10** and **Figure S11**.

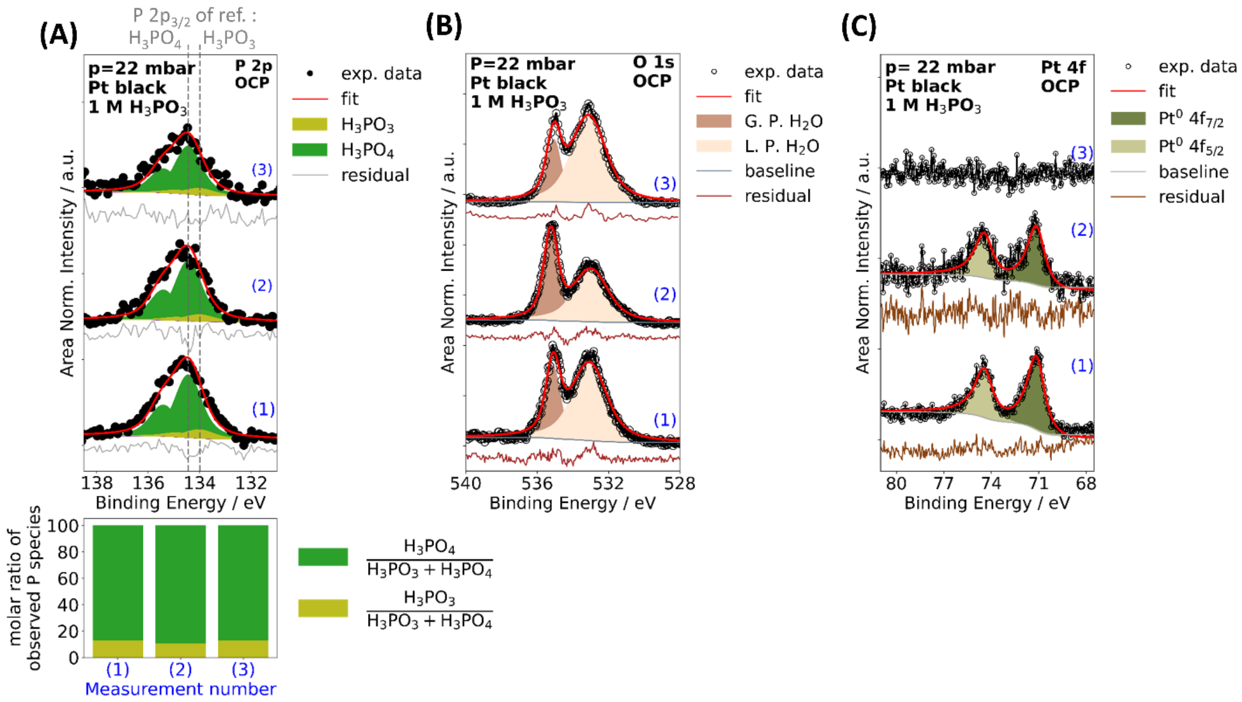

Figure S10. *In situ* "Dip-and-pull" AP-HAXPES data of 1 mol dm^{-3} (1 M) H_3PO_3 on Pt black: **(A)** P 2p, **(B)** O 1s, and **(C)** Pt 4f core levels. "G. P. H_2O" and "L. P. H_2O" in panel (B) represent: "gas phase H_2O" and "liquid phase H_2O", respectively. All measurements were performed at open circuit potential (OCP), at the pressure of 22 mbar, and incoming photon energy of 3 keV. Measurements (1) and (2) were performed at the Pt-electrode|aqueous H_3PO_3 interface, while measurement (3) was carried out at a slightly thicker electrolyte layer, where the signal arising from the electrode (i.e., the Pt 4f) could not be observed. Note that measurement (1) is the same measurement of the Pt black|1 mol dm^{-3} H_3PO_3 interface, discussed in the main text, and it is re-plotted for easier comparison with the additional measurements.

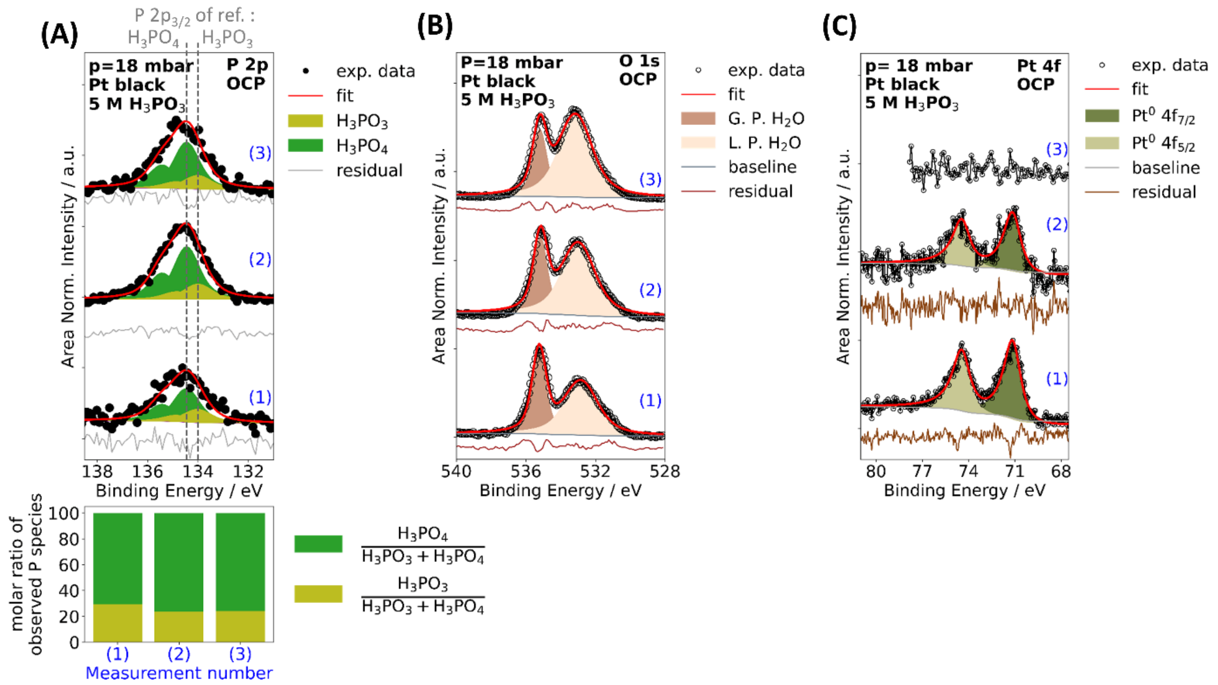

Figure S11. *In situ* "Dip-and-pull" AP-HAXPES of 5 mol dm^{-3} (5 M) H_3PO_3 on Pt black: **(A)** P 2p, **(B)** O 1s, and **(C)** Pt 4f core levels. "G. P. H_2O" and "L. P. H_2O" in panel (B) represent: "gas phase H_2O" and "liquid phase H_2O", respectively. All measurements were performed at open circuit potential (OCP), at the pressure of 18 mbar, and incoming photon energy of 3 keV. Measurements (1) and (2) were performed at the Pt-electrode|aqueous H_3PO_3 interface, while measurement (3) was carried out at a slightly thicker electrolyte layer, where the signal arising from the electrode (i.e., the Pt 4f) could not be observed. Note that measurement (1) is the same measurement of the Pt black|1 mol dm^{-3} H_3PO_3 interface, discussed in the main text, and it is re-plotted for easier comparison with the additional measurements.

As illustrated in **Figure S10** and **S11**, measurements conducted at the Pt-electrode|aq. H_3PO_3- electrolyte interface (labeled as measurement (1) and (2) in **Figure S10** and **S11**) show similar results of: **(i)** a high extent of oxidation of the H_3PO_3 electrolyte, **(ii)** exclusive presence of metallic Pt (i.e. $Pt^{(0)}$) at the interface, and **(iii)** a higher degree of H_3PO_3 electrolyte oxidation for the Pt black|1 mol dm^{-3} H_3PO_3 interface compared to Pt black|5 mol dm^{-3} H_3PO_3.

Furthermore, when comparing measurements performed at the Pt|aq. H_3PO_3 electrolyte interface (i.e., measurement (1) and (2)) to the experiment performed on a slightly thicker part of the electrolyte layer (i.e., measurement (3)), only a slight difference is observed between them. In both cases pronounced oxidation of aqueous H_3PO_3 can be observed. Note that for measurement (3), the electrolyte layer thickness could not be properly estimated via Eq. S1, since the signal corresponding to the Pt 4f core level of the electrode could not be measured. Nevertheless, considering that all of the measurement positions are located more than 0.5 cm above the surface of the bulk electrolyte in the beaker, it is likely that this spot possesses an electrolyte thickness of less than 50 nm, as previously suggested in Ref. [8].

Consequently, an increase in the local concentration of H_3PO_4 likely occurs at this measurement position (i.e. in the thin electrolyte layer of the formed meniscus), similar to what is observed in the other measurement positions, as discussed in the main text.

15. Comparison of the electrochemical characterization conducted in thin-film only configuration versus fully immersed electrode configuration.

It is necessary to confirm that the cyclic voltammogram (CV) shape on the 5 mol dm^{-3} H_3PO_3|Pt black electrode made with "Dip-and-pull" configuration is influenced mainly by the H_3PO_3 in the bulk electrolyte, as a large portion of the electrode is immersed in the bulk electrolyte compared to the thin film electrolyte part formed by "Dip-and-pull" process. This is to confirm the discussion in the main text of the CVs given in **Figure 4.D** and **4.E**. For this validation, cyclic voltammograms were performed with two configurations: "fully-immersed" configuration, in which electrochemical response will be arising only from the electrode immersed in the electrolyte, as well as with "thin-film-only" configuration, in which the voltammogram result will be emerging only from the on the electrode covered by the thin film of electrolyte (formed by the "Dip-and-pull" method).

To deconvolve the electrochemical response originating from the immersed electrode in contact with the bulk electrolyte and the electrochemical response arising from the thin film layer electrolyte (formed by the "Dip-and-pull" method), a modified Pt black electrode was used for the "thin-film-only" configuration experiment. This modified Pt black electrode has a lower part not covered with Pt black, rather consisting only of the un-sputtered non-conducting Si substrate, as shown in **Figure 12.A.** The "thin-film-only" configuration was achieved by performing the "Dip-and-pull" process to the point where all of the Pt black electrode is out of the bulk solution. The lower portion of the electrode, which consist only of Si, is still immersed in the electrolyte after the "Dip-and-pull" process to ensure a stable thin film formation. With this configuration, the electrochemical response measured in the thin-film-only configuration will only be arising from the Pt black in contact with the thin-film electrolyte and not from the bulk electrolyte. On the other hand, for the "fully-immersed" configuration the conventional "Dip-and-pull" method was still used, but some of the Pt black was still immersed in the bulk electrolyte and not fully retracted out of the solution. The scheme of the two measurement configurations is given in **Figure S12.B**.

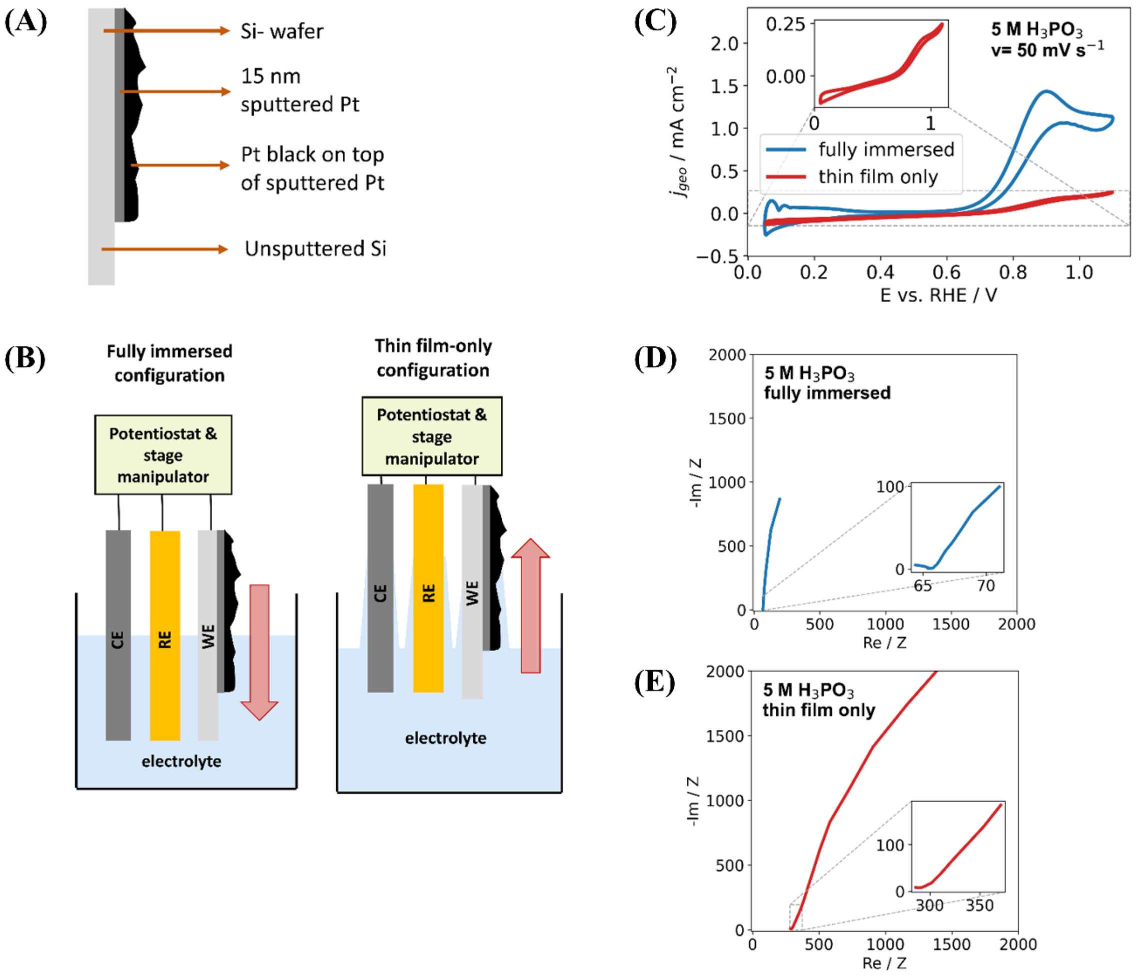

Figure S12. (A) Illustration of the working electrode used for the experiment: The top part of the working electrode is a Pt black electrode which is prepared on top of Pt sputtered Si substrate. The bottom part of the electrode is an un-sputtered Si substrate. **(B)** Two measurement configurations used in the experiment: "fully-immersed" working electrode configuration and "thin-film-only" configuration. **(C)** CV was recorded in 5 mol dm^{-3} H_3PO_3 with both measurement configurations. **(D), (E)** galvanostatic electrochemical impedance spectroscopy for determination of the cell resistance and IR compensation for the "fully immersed", and the "thin-film-only" configuration, respectively.

As shown in **Figure S12.C**, a much larger current was observed with the "fully-immersed" electrode configuration compared to the "thin-film-only" configuration, implying that in the CV presented in the main text, the majority of the current response originates from the electrode which is immersed in the bulk electrolyte compared to the thin film portion. Moreover, a lower current response and higher uncompensated ohmic resistance were observed with the aforementioned "thin-film-only" configuration (**Figure S12.D** and **Figure S12.E**). Additionally, the geometry of the thin film suggests the possibility of a strong inhomogeneity of the potential distribution during polarization along the meniscus height. As a result, a well-defined H_3PO_3 oxidation peak is absent in the "thin-film-only" configuration (see the inset graph of **Figure S12.C**).

16. References

(1) Green, C. L.; Kucernak, A. Determination of the Platinum and Ruthenium Surface Areas in Platinum−Ruthenium Alloy Electrocatalysts by Underpotential Deposition of Copper. I. Unsupported Catalysts. *J. Phys. Chem. B* **2002**, *106* (5), 1036–1047. https://doi.org/10.1021/jp0131931.

(2) Wei, C.; Sun, S.; Mandler, D.; Wang, X.; Qiao, S. Z.; Xu, Z. J. Approaches for Measuring the Surface Areas of Metal Oxide Electrocatalysts for Determining Their Intrinsic Electrocatalytic Activity. *Chem. Soc. Rev.* **2019**, *48* (9), 2518–2534. https://doi.org/10.1039/C8CS00848E.

(3) Sheng, W.; Myint, M.; Chen, J. G.; Yan, Y. Correlating the Hydrogen Evolution Reaction Activity in Alkaline Electrolytes with the Hydrogen Binding Energy on Monometallic Surfaces. *Energy Environ. Sci.* **2013**, *6* (5), 1509. https://doi.org/10.1039/c3ee00045a.

(4) de Groot, F.; Kotani, A. *Core Level Spectroscopy of Solids*, 0 ed.; CRC Press, 2008. https://doi.org/10.1201/9781420008425.

(5) Newberg, J. T.; Starr, D. E.; Yamamoto, S.; Kaya, S.; Kendelewicz, T.; Mysak, E. R.; Porsgaard, S.; Salmeron, M. B.; Brown, G. E.; Nilsson, A.; Bluhm, H. Formation of Hydroxyl and Water Layers on MgO Films Studied with Ambient Pressure XPS. *Surf. Sci.* **2011**, *605* (1–2), 89–94. https://doi.org/10.1016/j.susc.2010.10.004.

(6) Emfietzoglou, D.; Nikjoo, H. Accurate Electron Inelastic Cross Sections and Stopping Powers for Liquid Water over the 0.1-10 KeV Range Based on an Improved Dielectric Description of the Bethe Surface. *Radiat. Res.* **2007**, *167* (1), 110–120. https://doi.org/10.1667/RR0551.1.

(7) Shinotsuka, H.; Tanuma, S.; Powell, C. J.; Penn, D. R. Calculations of Electron Inelastic Mean Free Paths. X. Data for 41 Elemental Solids over the 50 EV to 200 KeV Range with the Relativistic Full Penn Algorithm: Calculations of Electron Inelastic Mean Free Paths. X. *Surf. Interface Anal.* **2015**, *47* (9), 871–888. https://doi.org/10.1002/sia.5789.

(8) Ali-Löytty, H.; Louie, M. W.; Singh, M. R.; Li, L.; Sanchez Casalongue, H. G.; Ogasawara, H.; Crumlin, E. J.; Liu, Z.; Bell, A. T.; Nilsson, A.; Friebel, D. Ambient-Pressure XPS Study of a Ni–Fe Electrocatalyst for the Oxygen Evolution Reaction. *J. Phys. Chem. C* **2016**, *120* (4), 2247–2253. https://doi.org/10.1021/acs.jpcc.5b10931.

(9) Axnanda, S.; Crumlin, E. J.; Mao, B.; Rani, S.; Chang, R.; Karlsson, P. G.; Edwards, M. O. M.; Lundqvist, M.; Moberg, R.; Ross, P.; Hussain, Z.; Liu, Z. Using “Tender” X-Ray Ambient Pressure X-Ray Photoelectron Spectroscopy as A Direct Probe of Solid-Liquid Interface. *Sci Rep* **2015**, *5* (1), 9788. https://doi.org/10.1038/srep09788.

(10) Favaro, M.; Jeong, B.; Ross, P. N.; Yano, J.; Hussain, Z.; Liu, Z.; Crumlin, E. J. Unravelling the Electrochemical Double Layer by Direct Probing of the Solid/Liquid Interface. *Nat Commun* **2016**, *7* (1), 12695. https://doi.org/10.1038/ncomms12695.

(11) Stoerzinger, K. A.; Favaro, M.; Ross, P. N.; Hussain, Z.; Liu, Z.; Yano, J.; Crumlin, E. J. Stabilizing the Meniscus for Operando Characterization of Platinum During the Electrolyte-Consuming Alkaline Oxygen Evolution Reaction. *Top Catal* **2018**, *61* (20), 2152–2160. https://doi.org/10.1007/s11244-018-1063-6.

(12) Stoerzinger, K. A.; Favaro, M.; Ross, P. N.; Yano, J.; Liu, Z.; Hussain, Z.; Crumlin, E. J. Probing the Surface of Platinum during the Hydrogen Evolution Reaction in Alkaline Electrolyte. *J. Phys. Chem. B* **2018**, *122* (2), 864–870. https://doi.org/10.1021/acs.jpcb.7b06953.

(13) Sugawara, S.; Tsujita, K.; Mitsushima, S.; Shinohara, K.; Ota, K. Simultaneous Electrochemical Measurement of Oxygen Reduction and Pt Oxide Formation/Reduction on Pt Nanoparticle Surface. *Electrocatal* **2011**, *2* (1), 60–68. https://doi.org/10.1007/s12678-010-0036-8.
